# Structural Basis for the ABO Blood-Group Dependence of *Plasmodium falciparum* Rosetting

**DOI:** 10.1371/journal.ppat.1002781

**Published:** 2012-07-12

**Authors:** Inès Vigan-Womas, Micheline Guillotte, Alexandre Juillerat, Audrey Hessel, Bertrand Raynal, Patrick England, Jacques H. Cohen, Olivier Bertrand, Thierry Peyrard, Graham A. Bentley, Anita Lewit-Bentley, Odile Mercereau-Puijalon

**Affiliations:** 1 Institut Pasteur, Unité d'Immunologie Moléculaire des Parasites, Paris, France; 2 CNRS URA 2581, Paris, France; 3 Institut Pasteur, Unité d'Immunologie Structurale, Paris, France; 4 CNRS UMR 3528, Paris, France; 5 Institut Pasteur, Protéopole, Plate-Forme de Biophysique des Macromolécules et de leurs Interactions, Paris, France; 6 Université de Reims Champagne Ardenne, URCA EA3798, Reims, France; 7 INSERM UMR-S 665, Institut National de la Transfusion Sanguine Paris, Université Denis Diderot, Paris, France; 8 Centre National de Référence pour les Groupes Sanguins (CNRGS), Institut National de la Transfusion Sanguine (INTS), Paris, France; Institute of Immunology and Infection Research, United Kingdom

## Abstract

The ABO blood group influences susceptibility to severe *Plasmodium falciparum* malaria. Recent evidence indicates that the protective effect of group O operates by virtue of reduced rosetting of infected red blood cells (iRBCs) with uninfected RBCs. Rosetting is mediated by a subgroup of PfEMP1 adhesins, with RBC binding being assigned to the N-terminal DBL1α_1_ domain. Here, we identify the ABO blood group as the main receptor for VarO rosetting, with a marked preference for group A over group B, which in turn is preferred to group O RBCs. We show that recombinant NTS-DBL1α_1_ and NTS-DBL1α_1_-CIDR1γ reproduce the VarO-iRBC blood group preference and document direct binding to blood group trisaccharides by surface plasmon resonance. More detailed RBC subgroup analysis showed preferred binding to group A_1_, weaker binding to groups A_2_ and B, and least binding to groups A_x_ and O. The 2.8 Å resolution crystal structure of the PfEMP1-VarO Head region, NTS-DBL1α_1_-CIDR1γ, reveals extensive contacts between the DBL1α_1_ and CIDR1γ and shows that the NTS-DBL1α_1_ hinge region is essential for RBC binding. Computer docking of the blood group trisaccharides and subsequent site-directed mutagenesis localized the RBC-binding site to the face opposite to the heparin-binding site of NTS-DBLα_1_. RBC binding involves residues that are conserved between rosette-forming PfEMP1 adhesins, opening novel opportunities for intervention against severe malaria. By deciphering the structural basis of blood group preferences in rosetting, we provide a link between ABO blood grouppolymorphisms and rosette-forming adhesins, consistent with the selective role of *falciparum* malaria on human genetic makeup.

## Author Summary

Rosetting, the capacity of infected red blood cells (RBCs) to bind uninfected RBCs, is a *Plasmodium falciparum* virulence factor. Rosetting is influenced by the ABO blood group, being less efficient with O RBCs. Although this preference may account for protection against severe malaria afforded by the O blood group, its understanding is fragmentary. We identify the ABO blood group as the main receptor for the rosetting Palo Alto VarO parasites, which display a marked preference for blood group A. Rosetting is caused by a sub-group of PfEMP1 adhesins. PfEMP1-VarO shares with other rosetting lines a specific NTS-DBL1α_1_-CIDR1γ Head region. We show that the Head region binds RBCs more efficiently than NTS-DBL1α_1_ and that ABO blood group polymorphisms influence binding of both domains. The 2.8 Å resolution crystal structure of the Head region reveals extensive contacts between the DBL1α_1_ and CIDR1γ domains, and shows structural features of the NTS-DBL1α_1_ hinge region essential for RBC binding. We localize the RBC-binding site to the face opposite to the heparin-binding site of NTS-DBL1α_1_ and document direct binding of the Head region to A and B trisaccharides These findings provide novel insights into the interactions established by malaria parasites with a prominent human blood group.

## Introduction

The ABO blood group system of carbohydrate antigen expression on the surface of human red blood cells (RBCs) is critically important in transfusion medicine. Several associations have been reported between the ABO blood group phenotype and relative risk of infectious diseases, including malaria [1]–[6]. In the case of *Plasmodium falciparum* malaria, recent studies have indicated that blood group O confers a protective effect against severe malaria [7]–[9]. The best-documented parasite determinant associated with the ABO blood group is rosetting, the capacity of infected RBCs to bind uninfected RBCs, which is consistently associated with severe malaria in African children [10]–[12] and is reduced inblood group O individuals [9], [12]–[16]. The hypothesis that group O protects against severe malaria by virtue of reduced rosetting has received strong support in a case-control study in Mali [9].

Although the ABO blood group preference of rosetting has been long known, understanding of its molecular basis is still fragmentary. Rosetting is caused by a sub-group of PfEMP1 adhesins encoded by the large *var* gene family. The extracellular region of PfEMP1 comprises multiple adhesion domains called Duffy Binding-Like (DBL) and Cysteine-Rich Interdomain Region (CIDR) [17]. DBL and CIDR domains are classified into different major classes (α to ε) and sub-classes by sequence criteria, while the *var* genes can be classified into specific subfamilies that possess distinctive upstream and downstream flanking regions [18]–[21]. Efforts to unravel the molecular basis of PfEMP1-mediated rosetting are complicated by the mosaic structure of the *var* genes and the population diversity of *var* repertoires [20], [22]. Nevertheless, the rosette-forming PfEMP1 adhesins described so far, namely IT4/R29 [23], Palo Alto 89F5 VarO [24], 3D7/PF13_0003 [25] and IT4/var60 [26], belong to a specific sub-group called groupA/UpsA *var* genes and, interestingly, all four present a specific DBL1α_1_-CIDR1γ double domain Head region [19]. Analysis of pseudo-rosettes formed on the surface of COS cells or baculovirus-infected insect cells expressing individual PfEMP1 domains have mapped the RBC adhesion region to the N-terminal DBL1α_1_ domain [23], [24], [26].

Here, we sought to understand the structural basis of the blood group preference in PfEMP1-VarO rosetting. We show that Palo Alto 89F5 VarO parasites bind to RBCs with a marked preference for blood group A compared to blood groups B and O. The binding preference of the PfEMP1-VarO N-terminal domain NTS-DBL1α_1_ (termed hereafter DBL1α_1_, as we have shown that NTS is a structural component of this domain [27]), and the DBL1α_1_-CIDR1γ double domain (called hereafter Head) mirror the ABO blood group preference of VarO parasites. Direct binding of the Head region to blood group trisaccharides was demonstrated by surface plasmon resonance. A more detailed blood group analysis showed that polymorphisms influence binding, with much stronger binding to subgroup A_1_ than to subgroup A_2_, and minimal binding to A_x_ (weak variant of A) RBCs. We determined the crystal structure of the double domain Head protein, the first structure of a multiple domain segment of PfEMP1, which reveals numerous interactions between the DBL1α_1_ and CIDRγ domains, and shows specific structural features of the CIDRγ class that differ from the published CIDRα class [28]. Importantly, we clarified the structure of the NTS-DBL1α_1_ hinge region that was lacking in our previous structural analysis of the DBL1α_1_-VarO domain [27]. We show that this region is surface-exposed and critical for RBC binding. The structural information obtained from the functional Head protein was used for computer docking and site-directed mutagenesis in order to localize the RBC-binding site. This was mapped to a specific region of the DBL1α_1_ domain, which is structurally conserved between different rosetting variants and is located on the face opposite to that of the major heparin-binding site. This work identifies the interaction with the ABO group as pivotal in rosetting and associates, for the first time, *P. falciparum* rosetting with the A subgroups, consistent with a contribution of virulent malaria to the selection of ABO blood group polymorphisms. The molecular description of the RBC-binding site provides novel perspectives for the development of preventive or therapeutic measures to combat severe malaria.

## Results

### VarO rosetting and ABO blood group preference

**Figure 1:**
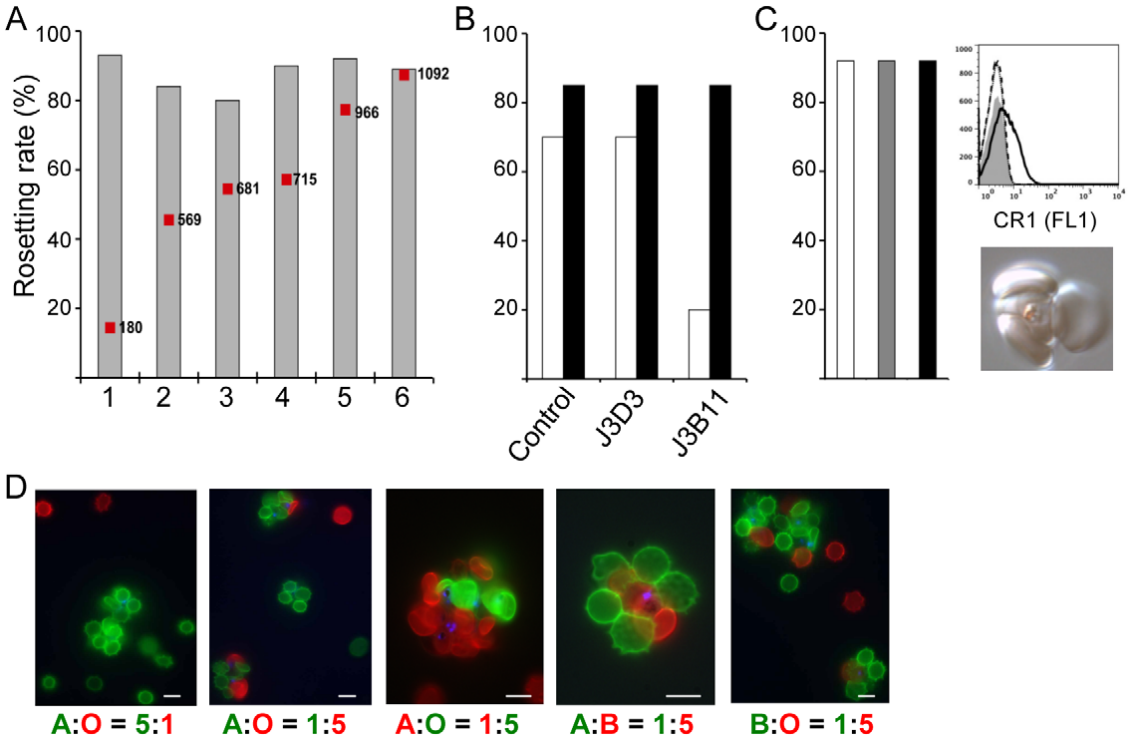
VarO rosetting is CR1 independent and ABO blood group dependent. (**A**) Rosetting rate of monovariant Palo Alto 89F5 VarO parasites cultivated in RBC samples displaying distinct CR1 copy number, which was assessed using biotinylated anti-CR1/CD35 mAb J3D3 (red squares). Rosetting rates (grey bars) were assessed at similar parasitemia for all cultures. Growth and re-invasion rates were similar for all donors tested. (**B**) Rosette disruption assay with anti-CR1 mAbs J3D3 (IgG1) and J3B11 (IgG1), and a mouse IgG1 isotype control. Rosette-enriched Palo Alto 89F5 VarO (black bars) and IT4/R29 (open bars) parasites cultivated in the same batch of RBCs were diluted in the presence of 10 µg.mL^−1^ antibody and incubated at 37°C for 30 min before assessment of rosetting rate. (**C**) Rosette formation of VarO-iRBC with recipient RBCs where CR1 was enzymatically cleaved. The graph shows the rosetting rate of untreated recipient RBCs (white), trypsin-treated recipient RBCs (grey) and chymotrypsin-treated recipient RBCs (black). The upper inset shows a histogram representation (CR1 expression level vs. cell count) of a typical CR1 flow cytometry detection assay using mAb J3B11: untreated RBCs (black curve); trypsin-treated RBCs (dotted curve); chymotrypsin-treated RBCs (dashed curve); background labelling (filled grey curve). The lower inset shows a typical VarO-iRBC rosette formed with trypsin-treated recipient RBCs. (**D**) ABO blood group preference of Palo Alto 89F5 VarO-iRBCs. Purified VarO-iRBCs were incubated in the presence of varying ratios of recipient A, B and O RBCs differentiated by labelling alternately with the lipophilic fluorescent probes PKH26 or PKH67. Images shown were at a ×40,000 or ×100,000 magnification. At the bottom of each panel, the scale bars correspond to 7 µm. The rosetting rate was evaluated by fluorescence microscopy. A representative result of at least three independent assays is shown.

VarO-mediated rosetting was dependent on the presence of a minimum of 5% human serum (Figure S1A), consistent with findings with other laboratory lines [29]–[32] and field isolates [33]. VarO rosetting is not CR1-dependent, as the rosetting rate was not correlated with the CR1 expression level on the recipient RBCs (Figure 1A), was unaffected by the anti-CR1 mAb J3B11 (which reduces rosetting of the R29 line [34]) (Figure 1B), and, importantly, was unchanged when CR1 was cleaved with trypsin or chymotrypsin (Figure 1C). Likewise, immunoglobulin binding, implicated in rosetting in some lines [32], [35], [36], apparently does not come into play in VarO rosetting, as VarO-iRBC rosetting was unimpaired in Ig-depleted human serum (Figure S1B) and no IgG or IgM binding could be shown on the surface of VarO-iRBCs(Figure S1C).

Incubation of VarO-iRBCs with varying ratios of recipient group A and group O RBCs, differentially labeled using lipophilic fluorescent probes PKH67 or PKH26, showed preferential binding to the former (Figure 1D). Blood group A was also preferred to blood group B but rosette formation was more efficient with group B than with group O RBCs (Figure 1D). This ABO blood group dependence, in particular the reduced binding to group O RBCs, is in line with previous observations with rosetting lines and field isolates [9], [13], [14], [16]. Although VarO rosettes could be readily disrupted by low concentrations of sulphated glycosaminoglycans such as heparin [37], they could not be disrupted by soluble blood group A or B trisaccharides used in the 20–40 mM range (data not shown), consistent with reports on other rosetting lines [13], [14].Because VarO rosettes were resistant to mechanical disruption, we could not test the capacity of trisaccharides to inhibit the reformation of rosettes as done for some rosetting lines [13], [14].

### RBC binding of soluble recombinant PfEMP1-VarO domains

**Figure 2:**
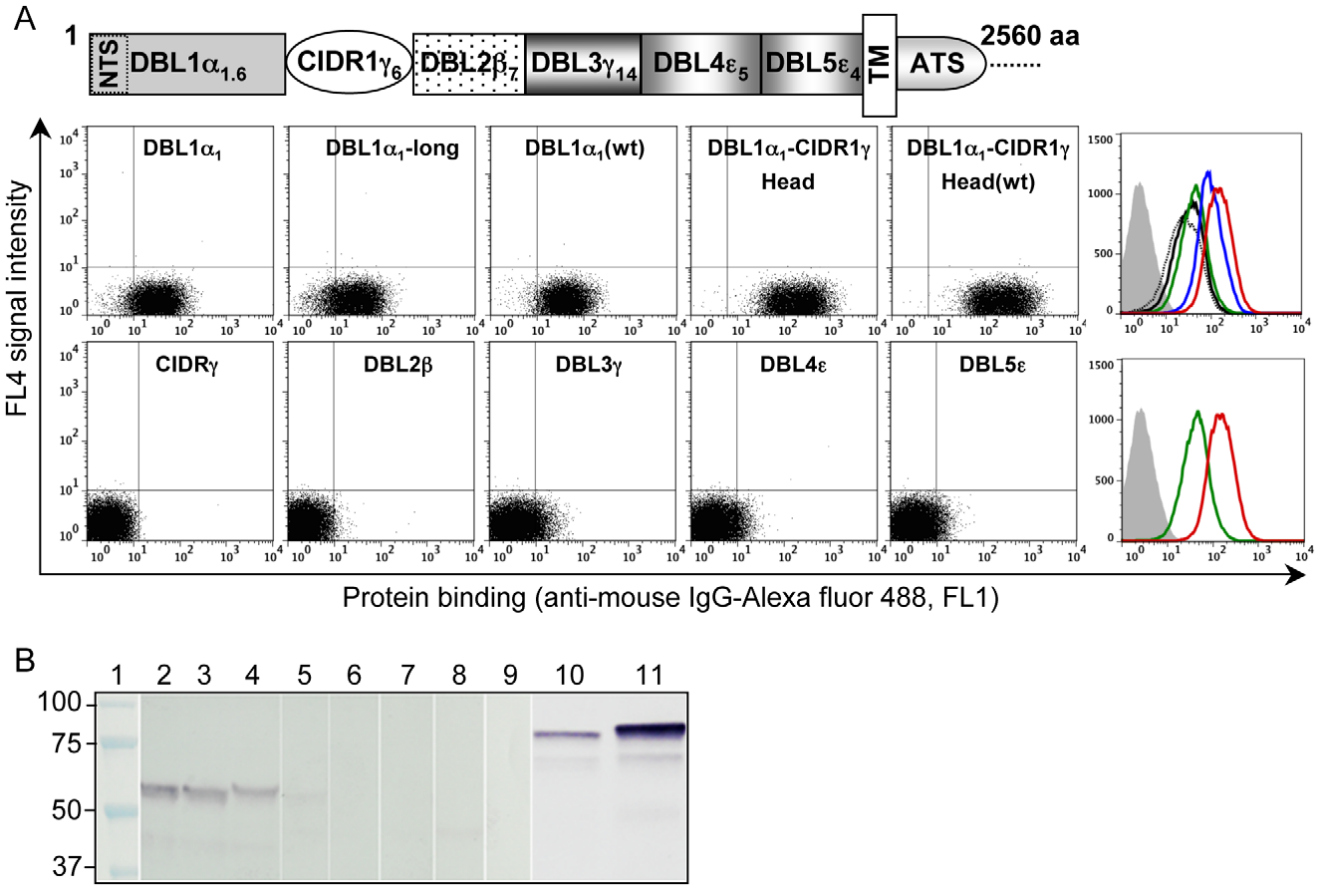
RBC binding capacity of soluble recombinant PfEMP1-VarO domains. A schematic representation of the PfEMP1-VarO domain organisation is shown in the upper part of the figure. The six individual recombinant domains and the Head were produced as soluble proteins in *E. coli* (see Figure S2). Binding to freshly collected blood group A_1_ RBCs was assayed at identical molar concentrations of recombinant protein and visualised using polyclonal mouse antibodies raised to the cognate domain and goat anti-mouse IgG labelled with Alexa fluor-488 or alkaline phosphatase. (**A**) Dot plot representation of flow cytometry analysis of the binding of DBL1α_1_, DBL1α_1_-long, DBL1α_1_(wt), Head, Head(wt), CIDR1 γ, DBL2β, DBL3γ, DBL4ε and DBL5ε. The right panels show histograms of representative results (FL1-MFI vs. cell counts) of RBC binding assays: background labelling - no protein added - (filled gray), DBL1α_1_(black), DBL1α_1_(wt)(green), DBL1α_1_-long (dotted black); Head (blue) and Head(wt)(red). (**B**) Representative immunoblot results of RBC binding: Molecular mass markers (lane 1), DBL1α_1_ (lane 2); DBL1α_1_(wt)(lane 3); DBL1α_1_-long (lane 4); CIDR1γ (lane 5); DBL2β (lane 6); DBL3γ (lane 7); DBL4ε (lane 8); DBL5ε (lane 9); Head (lane 10); Head(wt)(lane 11).

All six individual PfEMP1-VarO domains and the Head protein (DBL1α_1_-CIDR1γ, corresponding to residues 2–716) were produced as soluble recombinant proteins in *Escherichia coli* (Figures S2A and S2B). All proteins used in this study were monomeric, as judged by gel permeation chromatography (not shown). Correct protein folding was ascertained by CD spectroscopy, analytical ultra-centrifugation (Figure S2C) and the capacity to induce surface-reacting antibodies (Ab) (Table S1). RBC-binding capacity was monitored by immunoblot and flow cytometry using specific polyclonal sera at dilutions that did not disrupt VarO rosettes. To monitor binding of the Head protein, we used mAb G8.49, which reacted by ELISA with CIDR1γ (Figure S3). Equal molar amounts of protein were used in all assays. Binding was specific, as no signal was detected when the recombinant domain, the anti-VarO Abs or the secondary anti-IgG Abs were omitted. Of all six individual PfEMP1-VarO domains, only constructs containing the N-terminal DBL1α_1_ bound RBCs (Figures 2A and 2B). This confirmed previous findings with PfEMP1-VarO domains expressed on the surface of infected *Spodoptera frugiperda* (SF9) cells [24] and is consistent with reports by other groups [23], [38]. TheHead protein bound more efficiently than DBL1α_1_, the mean MFI being enhanced by about 1 log unit and the immunoblot signal consistently higher for the Head protein than for DBL1α_1_. Comparison of binding efficiency of DBL1α_1_ constructs of varying length showed essentially similar binding for an 18-Cys construct ending with the canonical Cys(11)xxCys(12) doublet (residues 2–471) and for the 20 Cys constructs (residues 2–487) with two additional Cys residues at the C-terminus (corresponding to the structurally complete DBL1α_1_ domain). Presence of a hexa-His tag at the C-terminus did not substantially modify binding (data not shown). As the various recombinant domains had their putative N-glycosylation sites removed (NxT/S mutated to NxA), we also produced recombinant DBL1α_1_ and Head with a wild-type (wt) coding sequence (DBL1α_1_(wt) and Head(wt), respectively). Seroreactivity was similar for the wt and the corresponding mutated constructs (Figure S4) but DBL1α_1_(wt) and Head(wt)bound slightly more efficiently than their mutated constructs (Figure 2).

RBC binding by mutated and wt constructs was heparin-sensitive and inhibited by rosette-disrupting mAbs (Figures S5A and S5B). Presence of human serum moderately enhanced RBC binding, an effect that was more marked for the Head constructs (2.9–4 fold increase) than the DBL1α_1_ constructs (1.4–2 fold increase) (Figures S5C and S5D). A similar enhancement was observed when foetal calf serum was added (Figure S5C). This indicates that the serum-enhancing activity is not species-specific and, as such, is different from the requirement of human-specific serum for VarO rosetting in human cells.

### ABO blood group preference of DBL1α_1_ and Head domains

**Figure 3:**
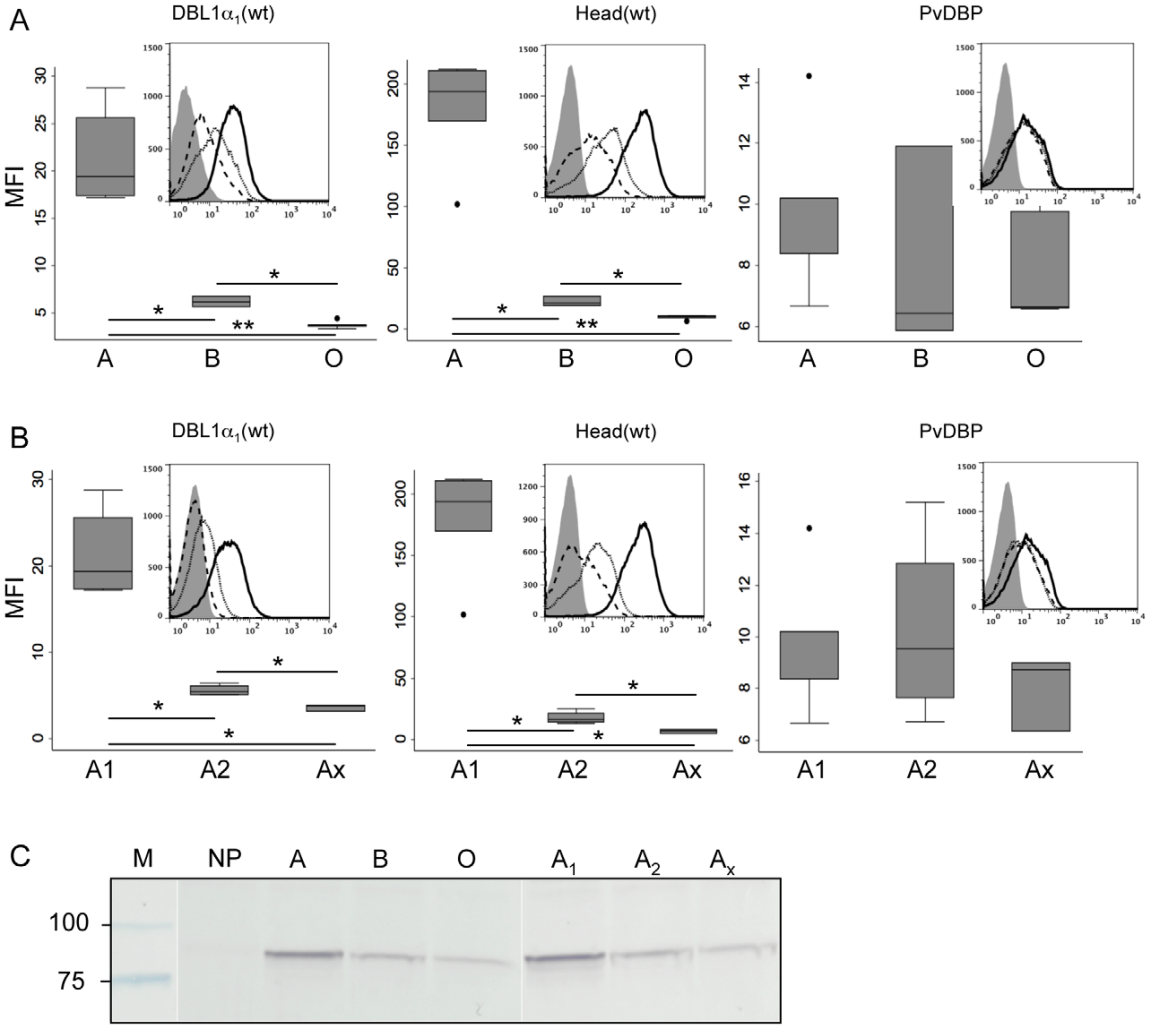
ABO Blood group preference of PfEMP1-VarO adhesion domains. Binding of DBL1α_1_(wt), Head(wt) and PvDBP recombinant domains to A, B or O RBCs and blood group A subgroups. Box plot representation of the mean fluorescence intensity (MFI) of protein binding to (**A**) a panel of cryo-preserved RBCs from reference blood donors [A (n = 5), B (n = 3) and O (n = 5)] and (**B**) blood group A subgroups [A_1_ (n = 5), A_2_ (n = 4) and A_x_ (n = 3)]. The boundaries of the boxes indicate the 25th and 75th percentiles, the line in each box indicates the median and the whiskers indicate the 10th and 90th percentiles. The outlying dots show values exceeding the 10th and 90th percentiles. The background reactivity of the antibodies to the different RBCs (no recombinant protein added) was 2±0.5 MFI Units. The results of Kruskal-Wallis nonparametric test are indicated by asterisks, as follows: *, P<0.05; **, P<0.01. The insets show for each binding experiment the flow cytometry histograms of a representative binding to an individual donor from each blood group. (**A**) binding to A (black curve), B (dotted curve) and O (dashed curve); (**B**) binding to A_1_ (black curve), A_2_ (dotted curve) and A_x_ (dashed curve); the background labelling is represented in grey filled curve. (**C**) Immunoblot analysis of Head(wt) binding to a representative individual donor from each blood group A, B, O, A_1_, A_2_ and A_x_; M, molecular mass markers; NP, no protein.

ABO blood group preference was explored for DBL1α_1_, DBL1α_1_(wt), Head and Head(wt) using a panel of RBC donors. All four proteins bound more efficiently to blood group A (N = 5) than to blood group B (N = 3) RBCs and, in turn, to blood group B more efficiently than to blood group O (N = 5) RBCs (Figure 3A and data not shown). This indicates that the terminal α-1,3-linked N-acetylgalactosamine (GalNAc) of blood group A and, to a lesser extent, galactose (Gal) of blood group B are key determinants of the interaction. The ABO blood group dependence was specific as no such dependence was observed for binding of the *P.vivax* Duffy Binding Protein PvDBP, known to bind Duffy-DARC on the RBC surface (Figure 3A).

We next explored binding to subtypes of group A RBCs, which differ mainly in the quantity of the terminal α-1,3-linked N-acetylgalactosamine (GalNAc) antigen displayed: subgroup A_1_ RBCs express up to four times as many A epitopes as subgroup A_2_, while A_x_ (weak variant of A) RBCs express very low amounts of terminal (GalNAc) [39]. Binding of DBL1α_1_(wt) and Head(wt)was subgroup-dependent, with binding to A_1_ (N = 5) being substantially higher than binding to A_2_ (N = 5), while binding to A_x_ (N = 3) was minimal (Figure 3B). Similar results were obtained when binding was monitored by immunoblot assays (Figure 3C). Binding strongly correlated with the amount of A antigen displayed on the recipient cell (Spearman coefficient correlation, rho = 0.96 (p<0.0001) and rho = 0.93 (p<0.0001) for DBL1α_1_(wt) and Head(wt) respectively) (Figure S6A). Furthermore, when A_2_ RBCs were treated with α-N-acetylgalactosaminidase, which cleaves the GalNAc groups, binding was reduced even further (data not shown). Binding showed an inverse relationship to the amount of H antigen displayed on the RBC (Spearman correlation coefficient, rho = −0.67 (p = 0.001) and rho = −0.70 (p = 0.0006) for DBL1α_1_(wt) and Head(wt)respectively) (Figure S6B). Levels of binding to A_2_ and B RBCs, which displayed similar amounts of H antigen, were similar. Consistent with this, removal of the terminal galactose from blood group B RBCs by treatment with galactosidase reduced their binding to DBL1α_1_(wt)(data not shown).

**Figure 4:**
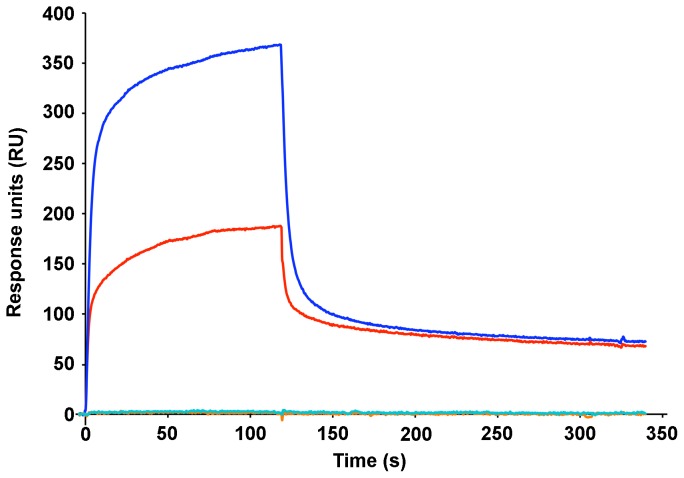
Direct binding to blood group trisaccharides assayed by surface plasmon resonance. Representative real-time association and dissociation profiles corresponding to the injection of the Head(wt) region (175 nM) over trisaccharide A (blue) or trisaccharide B (red) conjugated BSA. Premixing the Head(wt) region with a 2-fold excess of heparin resulted in no detectable binding to either conjugate (cyan and orange profiles for trisaccharide A- and trisaccharide B-BSA, respectively).

Direct binding of the recombinant Head(wt)domain to the blood group A and B trisaccharides was demonstrated using a surface plasmon resonance assay with covalently immobilized trisaccharide-conjugated-Bovine-Serum-Albumin as ligands. In line with the observations made on red blood cells, the Head (wt) region appears to bind more efficiently to group A than to group B conjugates (Figure 4). This was consistently observed across a large range of protein concentration (Figure S7). Furthermore, pre-mixing Head(wt) with a 2-fold excess of heparin totally abolished binding to both blood group BSA-conjugated tri-saccharides (Figure 4).

Together, these data show that binding is influenced by the amount and type of terminal glycan displayed on the RBC surface and is heparin-sensitive. The N-terminal DBL1α_1_ domain was identified as the minimal binding unit of the ABO blood group and the presence of CIDR1γ enhanced binding. In order to understand the molecular basis of these interactions, we solved the crystal structure of the double domain Head protein, since we had already determined the structure of the DBL1α_1_ domain [27].

### Crystal structure of the Head protein

The DBL1α_1_ structure we previously determined [27] was cleaved after residue 69, which turned out to abolish RBC binding (Figure S8, lane 2). The Head construct studied here, however, was prepared with the native, intact sequence. The Head crystallised only in the presence of heparin, as found earlier for DBL1α_1_, with the best crystals diffracting to 2.8 Å resolution. The Head structure (residues 12–487) was solved by molecular replacement and the polypeptide chain in the final model was traced for the entire sequence, with the exception of the first 18 N-terminal residues (see Table 1 for refinement statistics).

**Table 1:** Crystallographic data and refinement statistics.

**DIFFRACTION DATA**	
Space group/cell (Å)	C2/a = 157.9, b = 78.07, c = 46.98, β = 102.89°
Wavelength (Å)	0.98011
Resolution limits^a^ (Å)	40-2.8 (2.85-2.8)
R_merge_ a (%)	24.5 (1.634)
R_pim_ a ^,^ b (%)	11.3 (0.804)
No. unique reflections^a^	39457 (1550)
Mean((I)/σ(I))^a^	7.2 (0.8)
Completeness^a^ (%)	97.0 (84.7)
Multiplicity^a^	5.5 (4.7)
**REFINEMENT**	
Resolution^a^ (Å)	40.1 - 2.8
No. of reflections^a^	39457
No. test set reflections	2007
R_work_/R_free_ a (%)	21.5/25.5 (26.2/31.8)
No. of protein/ion/water atoms	5676/22/32
r.m.s.d. bonds(Å)/angles (°)	0.010/1.24
B-Wilson/B-average	67.1/76.5
Ramachandran plot	
preferred/allowed/outliers (%)	91.5/7.6/0.9

a Parenthesis refer to the highest resolution bin, 2.85-2.8 Å.

b Multiplicity-weighted R_merge_ [71].

**Figure 5:**
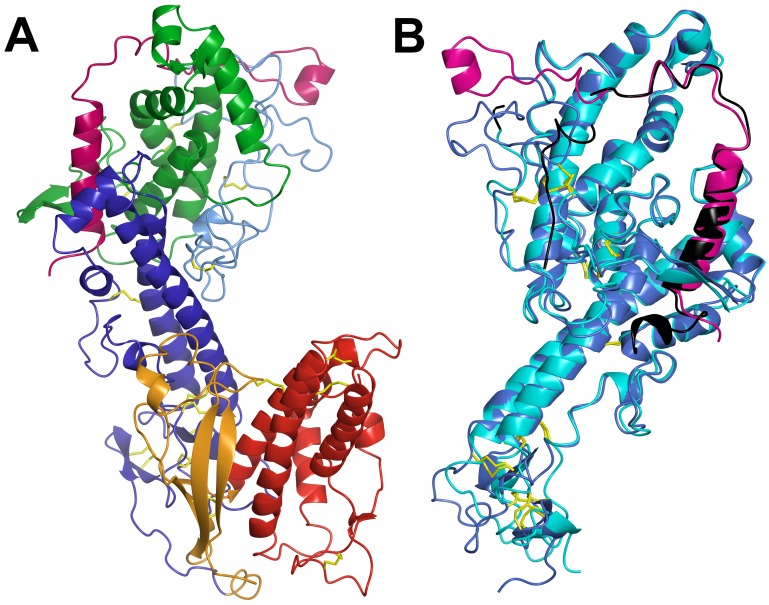
Structure of the DBL1α_1_-CIDR1γ VarO-Head region. (**A**) Overall view of the structure of the Head region with subdomains coloured as in Figure 6. (**B**) Superposition of the DBL1α_1_ structure determined earlier [27] (in cyan) upon the DBL1α_1_ domain in the Head structure (in blue). The NTS region is in black for the earlier cleaved structure and in mauve for the Head structure. The view is rotated 180° from (**A**).

**Figure 6:**
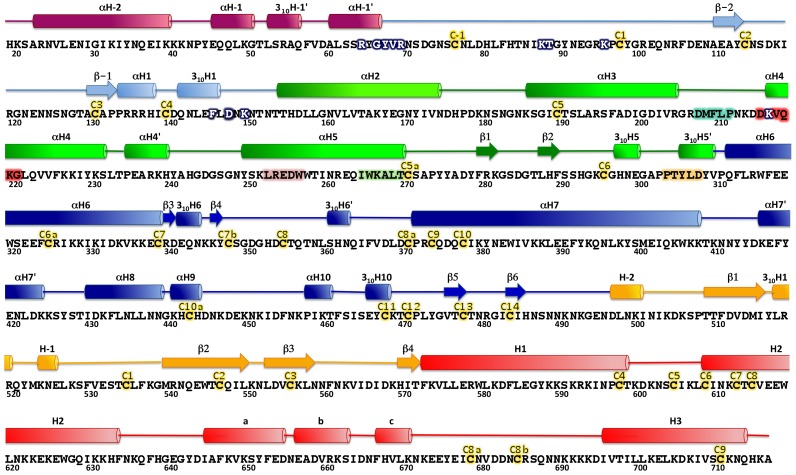
Sequence and secondary structure of DBL1α_1_-CIDR1γ VarO-Head region. The sequence is indicated in single letter code. Cylinders represent helices and arrows represent β strands. NTS is mauve; DBL1α_1_ subdomains 1, 2, and 3 are light blue, green, and blue, respectively, while CIDR1γsubdomains 1 and 2 are orange and red, respectively. Cysteines, given by their canonical nomenclature, are in yellow. DBL1α_1_ helices up to αH8 without primes (′) refer to helices common to all known DBL structures. Mutations that affect RBC binding are highlighted as white letters on a dark blue background. The sequences corresponding to the PolV tags are shown with backgrounds cyan (PolV1), pink (PolV2), green (PolV3) and orange (PolV4). Motif H3 is shown in red.

The overall shape of the Head is rather compact, with the CIDR1γ domain folding back upon the DBL1α_1_ domain, thus burying a significant surface at the interface (Figure 5A). The structure of the Head protein allows a detailed description of all segments that were missing in our previous analysis of the single DBL1α_1_ domain, in particular the stretch of residues 53 to 71 and 83 to 94 (Figure 5B). The NTS region contains two more helices, followed by a long surface-exposed loop that includes canonical cysteines Cys(−1) and Cys(1) of subdomain 1 (Figure 5B and 6). An antiparallel β-sheet then connects to αH1 at the conserved PPR motif, which is very close to the previous DBL1α_1_ structure. In the latter structure, we were able to trace only a short peptide D72-F82 in the hinge region, which has a different conformation to that of the intact Head protein analysed here. The structure of the major heparin-binding site is essentially unchanged and all of the typical DBL motifs of subdomain 2 (in green) and part of subdomain 3 (in blue) are very close in the Head and the single domain DBL1α_1_ (Figure 5B). Importantly, the structure of the PEXEL-like sequence of NTS is identical to that observed earlier [27], thus confirming that this sequence is buried and important for the stability of the protein. There are also differences in the region in contact with the CIDR1γ domain. The C-terminal moiety of helix αH6 and the N-terminal region of helix αH7, are displaced by up to 14 Å, while loop C357-D372 (including helix αH6′) follows a different path. The RMS difference between the DBL1α_1_ Cα coordinates of the Head and single domain structures is 2.9 Å for the complete domain, but only 1.3 Å when the NTS-DBL1α_1_ junction and the C-terminal region in contact with CIDR1γ are excluded. The density connecting DBL1α_1_ and CIDR1γ is rather poor and allowed modelling the polypeptide backbone atoms only. From D504 onwards, however, the electron density becomes clear and the rest of the CIDR1γ domain could be traced unambiguously.

**Figure 7:**
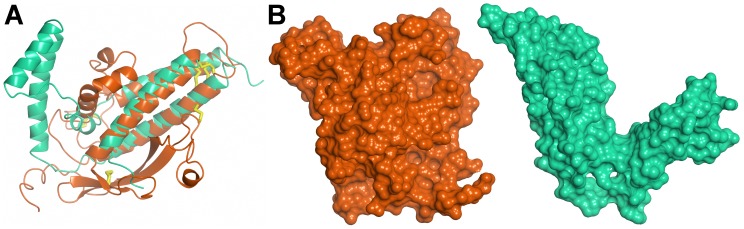
Structure of the CIDR1γ-VarO domain. (**A**) Superposition of the CIDR1γ -VarO domain (orange) upon the CIDRα-MC179 domain (turquoise). (**B**) Space-filling representation of CIDR1γ -VarO and CIDRα-MC179 domains.

The CIDR1γ-VarO domain (220 residues) forms a rather compact structure, in contrast to the CIDR1α-MC179 structure [28], which lacks the N-terminal part of the domain. In CIDR1γ-VarO, this region (residues 496–570) contains a twisted antiparallel β-sheet composed of four strands (β1-4) as well as three short α-helices (H-2, 3_10_H1, H-1) (Figure 5A and Figure 6), forming an N-terminal subdomain. The latter part of CIDR1γ is largely α-helical, with a pattern of helices similar to that of MC179, though helix **a** is shorter and helices **b** and **c** together span the length of helix **b** in MC179. The relative position of the three-helix bundle H1, H2, H3 and helices **a**, **b**, **c** is, however, quite different, giving CIDR1γ-VarO a much more compact shape (Figures 7A and 7B). The first Cys residue of CIDR1γ-VarO (C534, canonical Cys(1)) forms a disulfide bond with C614 (canonical Cys(8)) in helix H2. In our construct, CIDR1γ-VarO contains 11 Cys residues (31 for the entire Head protein) and thus there must be at least one free sulfhydryl group per molecule. The protein was therefore purified in the presence of 25 mM cystamine, which prevented the formation of covalent dimers. All Cys residues are engaged in disulfide bridges except one free Cys (C604, canonical Cys(5) of CIDR1γ) (Table S2). This free Cys is indeed quite exposed, but the electron density is not sufficiently well defined to show a covalent modification. The MC179 protein included 17 additional residues at the C-terminal end, including C168 (canonical Cys(10)), which paired with canonical Cys(5) [28]. In the VarO sequence there is a Cys doublet (C722 and C723) in the corresponding site and since it was not obvious which of the two would form a disulfide with Cys(5), we terminated our construct upstream of it at residue 716. The polypeptide chain ends in close proximity of C604 (canonical Cys(5)), however, and could conceivably form a disulfide bridge. We have been able to express a longer Head construct ending at V787 in soluble form but did not succeed in obtaining crystals.

### Analytical ultracentrifugation

The crystals were prepared in the presence of heparin, which, although not visible in the electron density, may have induced the domains to come together or interfered with any dimer formation. We therefore compared the overall shape of the individual domains and the double domain using analytical ultracentrifugation(AUC) in the absence of heparin. When the hydrodynamic characteristics of the double domain were calculated from the crystal coordinates [40], [41], the results showed that the shape of the double domain in solution was very similar to that found in the crystal. Furthermore, the overall shape of the isolated CIDR1γ domain in solution corresponds to that in the crystal structure of the double domain (Table S3). Both the CIDR1γ and the Head protein are monomeric in solution and in the crystal unit cell, as was the case for the single DBL1α_1_ domain [27].

### The blood group A binding site of DBL1α_1_

Given the preference of VarO iRBC, as well as the DBL1α_1_ domain and the Head, for binding to blood group A, we sought to localize its binding site in the two recombinant proteins. We initially used the collection of mutant constructs used for defining the heparin-binding site [27] (Table 2). Mut1 and Mut3, whose affinity for heparin is reduced by more than 100 fold, bind to RBCs, whereas Mut2 and Mut4, whose heparin binding is unaltered or only slightly affected, do not bind (Figure S9A), indicating that the heparin-binding and RBC-binding sites do not overlap.

**Table 2:** List of recombinant DBL1α_1_, Head and mutant domains produced in *E. coli*.

Name	Residues	Mutations	His6-tag	RBC binding
	(No Cys)			
DBL1á_1_-long_His_	2-487 (20)	#	Yes	++++
Mut K87	2-487 (20)	# K87	Yes	++++
DBL1á_1_-long	2-487 (20)	#	NO	+++
Mut10*	2-487 (20)	# G66I Y67E V68G	NO	No binding
DBL1á_1His_	2-471 (18)	#	yes	++++
DBL1á_1_	2-471 (18)	#	NO	+++
Mut0*	2-471 (18)	# K87	NO	++
Mut1*	2-471 (18)	# K87 K20 K32 K40	NO	++
Mut2*	2-471 (18)	# K87 K95 K166 K179	NO	No binding
Mut3*	2-471 (18)	# K87 K423 K424 K451 K456	NO	++
Mut4*	2-471 (18)	# K87 K226 K227 K230	NO	No binding
Mut5*	2-471 (18)	# K87 K404 K407 K410	NO	++
Mut6*	2-471 (18)	# K87 K117 K213	NO	++++
Mut7*	2-471 (18)	# K87 K379 K393	NO	++++
Mut8	2-471 (18)	# K87 R64 Y67 R69	NO	No binding
Mut9	2-471 (18)	# K87 T88 Y90 E92	NO	+
DBL1á_1_(wt)	1-471 (18)	Wild type sequence	Yes	++++
Mut14	1-471 (18)	R64	Yes	++
Mut15	1-471 (18)	T88	Yes	+
Mut16	1-471 (18)	K87	Yes	++
Mut17	1-471 (18)	K95	Yes	No binding
Mut18	1-471 (18)	D147 K149	Yes	+
Mut19	1-471 (18)	F145 K216	Yes	No binding
Head(wt)	1-716 (31)	Wild type sequence	Yes	+++++
Head	2-716 (31)	#	NO	+++++

# N-glycosylation sites mutated (NxT/S to NxA).

*: Mutants described in [27]. All the residues were mutated in Ala (A) except for Mut10.

In some protein preparations, spontaneous cleavage of the polypeptide occurred after residue R64 or R69 upon storage (depending on the protein). Detailed analysis showed that only the full-length protein retained the ability to bind (Figure S8, lane 1), thus indicating that the integrity of a surface-accessible region of the NTS moiety was essential for RBC binding. This is the region with a large difference between the crystal structures of the intact Head and the cleaved, single DBL1α_1_ domain. We therefore used the Head structure to guide additional site-directed mutagenesis. Mutation of residues from the hinge region between NTS and DBL1α_1_ (Mut 10, 11,12 and 13, see Table 2 and Figure S9B) disrupted binding, confirming its essential structural role.

**Figure 8:**
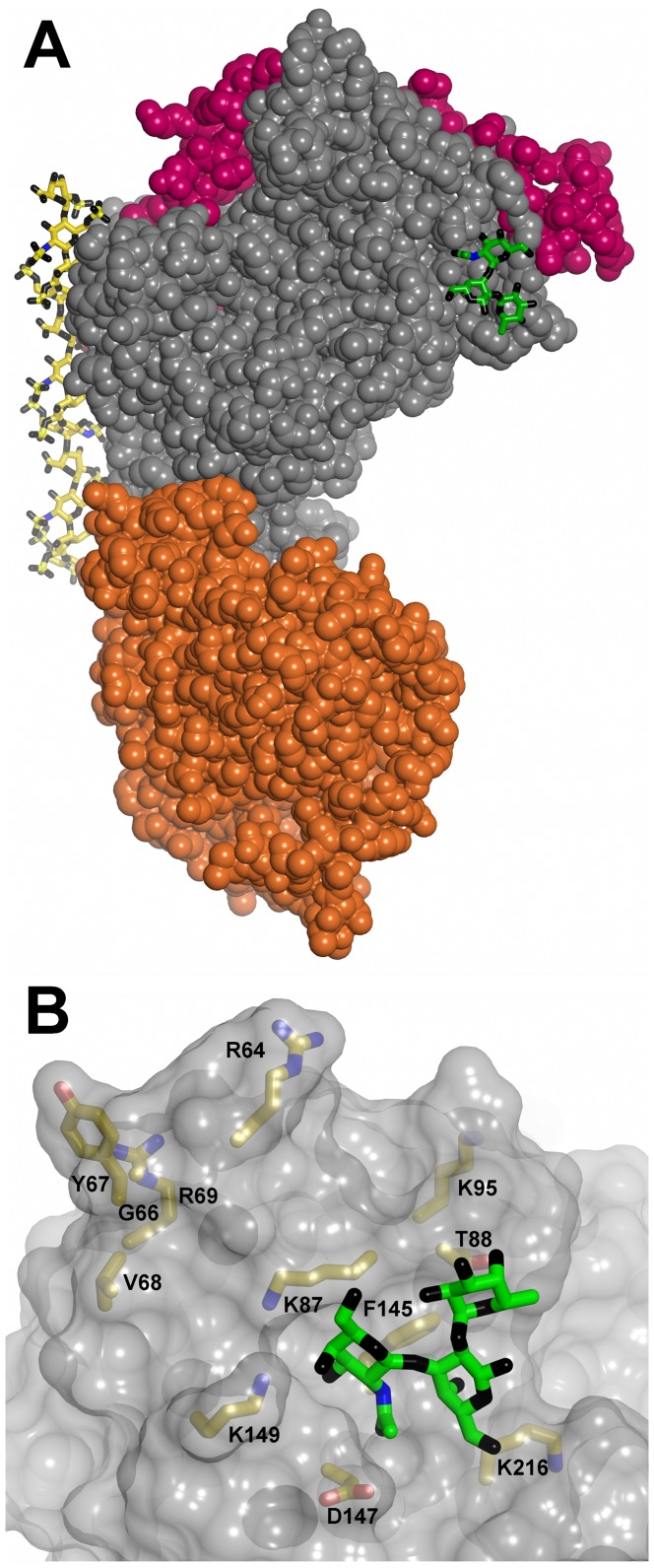
Localisation of the RBC binding site. (**A**) An overall view of the Head in a space-filling representation (NTS region in mauve, DBL1α_1_ domain in grey, CIDR1γ domain in orange) with the docked blood group A trisaccharide (green- carbon, black - oxygen and blue - nitrogen) and heparin (carbons in yellow) molecules in stick representation. (**B**) Detail of the RBC-binding site with side chains whose mutations affect binding highlighted (carbons in yellow, nitrogens in blue and oxygens in red).

We used computer docking to localize the potential binding site(s) of the terminal trisaccharide of blood group A within the Head protein. The lowest energy solutions of the docked trisaccharide were very tightly clustered about a site that included residues K87, T88, D147 and K149, corresponding to the region that is significantly different in the cleaved DBL1α_1_ domain (Figures 5B and 8). We therefore prepared a series of single or double mutants exploring this region of the structure. Because of the poor yields of the Head protein, these mutants were derived from the single DBL1α_1_ domain. This identified several key residues: K95 (Mut17), F145, K216 (Mut19), whose mutation completely disrupted binding, and T88 (Mut15) and D147, K149 (Mut18), whose mutation reduced binding by more than 90%. Single mutations,R64 (Mut14, localized within the NTS domain) and K87 (Mut16, located in the hinge region), reduced RBC binding by 50% (see Figure S9C). Overall, this demonstrates that the site predicted by computer docking was indeed critical for binding. The binding site includes the NTS segment and residues from subdomains 1 and 2 (Figure 6). Significantly, this region is conserved between rosetting strains and, moreover, is located on the face opposite to the major heparin-binding site (Figure 8A).

## Discussion

Our results identify the ABO blood group as the major VarO rosetting receptor on the host RBC and show that the presence of the CIDR1γ domain to form the Head region results in enhanced RBC binding, mimicking the ABO blood group preference of VarO rosetting. The crystal structure of the Head (DBL1α_1_-CIDR1γ) allowed mapping of the RBC-binding site to a structurally conserved region of rosette-forming PfEMP1 variants, involving residues from subdomains 1 and 2, with contributions from the neighbouring NTS-DBL1α_1_ hinge region. The RBC-binding site is distal to the heparin-binding site, indicating that although heparin prevents binding of the adhesion domain to the RBCs, it does not directly compete with the ligand-receptor interaction to prevent rosette formation.

Previous analyses of interactions at play in VarO rosetting showed that none of the common receptors of *P. falciparum* cytoadherence, such as CD36, ICAM-1, CSA, nor other potential receptors (VCAM-1, HABP1, CD31/PECAM, E-selectin, Endoglin, CHO receptor “X”, and Fractalkine) were implicated in the binding of RBC to VarO-iRBCs [42], [43]. We show here that CR1/CD35, shown to be a receptor for some rosetting lines (including R29 [23], [34]), is not involved in VarO rosetting. VarO rosetting shares with other rosetting lines three generic characteristics, namely an extreme sensitivity to sulphated glycosaminoglycans, the need for human serum and a marked ABO blood group preference characterised by reduced binding to group O RBCs. Our data indicate that the major determinant affecting VarO rosetting efficiency is indeed the ABO blood group. We explored VarO-iRBC binding characteristics using a monovariant culture of the Palo Alto 89F5 clone, in which >90% of the iRBCs were positively selected to express PfEMP1-VarO [24]. VarO-iRBCs preferentially bind to blood group A compared to blood group B, which itself is preferred to blood group O. An identical blood group preference profile was observed with both the DBL1α_1_ and Head proteins.

Our dissection of blood group A preference using the recombinant domains provides, for the first time, a link between rosetting and common group A polymorphisms. The difference between the A_1_ and A_2_ subgroups is mainly quantitative, with A_2_ RBC displaying 4–5 times fewer blood group A determinants than A_1_ [44], although they have some qualitative differences as well [45], [46]. Our observation that DBL1α_1_ hardly bound A_x_ RBCs is a strong indication that copy number variation of the terminal glycan is the main cause of differences in the binding behaviour between the three subgroups. Binding to A_2_ was similar in intensity to binding to blood group B. As the A_2_ RBCs expressed about 5-fold more H antigen than the B RBCs (Figure S6), we conclude that the terminal α-1,3-linked N-acetylgalactosamine (GalNAc) of group A is preferred to the terminal galactose (Gal) of group B. This was further documented by surface plasmon resonance assays, in which we explored binding to trisaccharide-BSA conjugates immobilized on the surface of a sensor chip. Binding to trisacharide A was more efficient than to trisaccharide B (Figure 4), with apparent affinities in the micromolar range. This blood group preference is fully consistent with the VarO-IRBCs blood group preference although it should be mentioned that BSA-conjugates sub-optimally mimic the RBC-displayed blood group saccharides, which present multiple branched saccharides or tandem copies of the blood group determinant.

**Figure 9:**
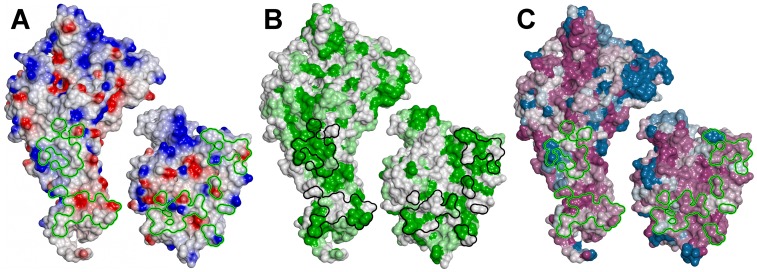
Interface between the DBL1α_1_ and CIDR1γ VarO domains. The two domains are represented with the contact surface, normally buried in the interface, facing the viewer (**A**) Electrostatic surface (red - negative and blue - positive; the scale is from −0.046 to 0.051 kT.e^−1^); (**B**) hydrophobic surface (green - hydrophobic, white - hydrophillic); (**C**) residue conservation across DBL1α_1_-CIDR1γ PfEMP1 proteins (from dark purple - highly conserved to blue - variable). The outline of the contact surface is shown as a green and black line.

The structure of the DBL1α_1_-CIDR1γ Head reported here is the first crystal structure of a multiple domain from PfEMP1, as well as of a complete CIDR1γ domain. The structure of the DBL1α_1_ domain published earlier [37] is confirmed and we further show that the CIDR1γ-VarO domain forms extensive contacts with the DBL1α_1_ region (Figures 9A and 9B). The interface between the DBL1α_1_ and CIDR1γ domains is stabilised by several charged interactions (D367-D654, N381-R518, E389-Y521, Y437-K550) and, furthermore, a significant hydrophobic surface on DBL1α_1_ is buried within this interface. A significant part of the CIDR1γ accessible surface (1367A^2^ of a total of 12477A^2^) is buried within the interface, including residues from its N-terminal half, as well as from the **a–b–c** helical motif (Figure 9). The interface is also highly conserved within the DBL1α_1_-CIDR1γ family of PfEMP1 adhesins (Figure 9C).

The three-dimensional structure of the Head allows a definitive assignment of interdomain boundaries. Thus the boundary between DBL1α_1_ and CIDR1γ domains lies within a rather flexible linker around residue 490, indicating that the DBL1α_1_ domain terminates after the disulphide Cys13–Cys14 (residues C477–C483of PfEMP1-VarO sequence). The CIDR1γ domain is more compact than the partial CIDRα structure published by [28], not only in our double-domain crystal structure but also in solution as a single domain (Figures 7 and S10). This difference could be due to a number of reasons: CIDR1γ -VarOcontains additional 98 residues at the N-terminus missing in the MC179 construct (which also lacked the preceding DBL1α domain) [28], the extensive contacts between DBL1α_1_ and CIDR1γ, or the dimer formation in the case of MC179. The two CIDR domains are of different sequence classes, γ for VarO and α for MC179. Moerover, the latter binds CD36, which is not the case for VarO [42], [43]. Both CIDR classes share a conserved arrangement of Cys and Trp residues in most of their sequence, with an additional disulphide (canonical Cys(8a)–Cys(8b)) in the γ class. The sequences are, however, much less well conserved in loop regions connecting both the helices and the subdomains, which could be another reason for the structural differences observed.

The similarity between DBL and CIDR domains in their general architecture, noted earlier [28], is also present in the VarO structure. Superposition of the CIDR1γ -VarO domain upon the DBL1α_1_-VarO domain matches not only helices H1, H2 and H3 (CIDR1 γ) to αH6, αH7 and αH10 (DBL1α_1_), but also helices **a** and **b** (CIDR1γ) to αH8 and αH9 (DBL1α_1_). Furthermore, strands β1 and β2 of CIDR1γ lie quite close to strands β-1 and β-2 of DBL1α_1_ (Figure S10). The similarity extends to the disulfide pattern as well, as suggested earlier [28]: canonical disulfides Cys(10)–Cys(11) and Cys(7)–Cys(9) of DBL1α_1_ overlap with disulfides Cys(7)–Cys(9) and Cys(4)–Cys(6) of CIDR1γ, respectively, while disulfide Cys(5)–Cys(10) of CIDR1γ lies close to Cys(8)–Cys(12) of DBL1α_1_. Indeed, the N-terminal half of CIDR could be classified as equivalent to subdomain 1 of DBL domains, while the second, helical domain of CIDR corresponds more closely to subdomain 3.

In the MC179 structure, the region implicated in CD36 binding [28] lies near the N-terminal end of helix **b**. In the CIDR1γ -VarO structure, the equivalent region corresponds to the **b–c** connecting loop, which faces the H1–H2–H3 helical bundle. Within the triplet S662-I663-D664 of VarO, corresponding to the critical residues E108-I109-K110 of MC179, S662 and I663 are buried by H1 and D664 forms a salt bridge with K591 from H1. Interestingly, S662 and I663 are highly conserved among CIDRγ sequences, suggesting that this loop may have a common conformation in this domain class. If these residues were implicated in CD36 binding, they would be poorly accessible in the rosetting strains of PfEMP1, which do not bind CD36 [43].

The structure of the Head provides critical information about the RBC-binding site. Computer docking and site-directed mutagenesis localized a blood group A binding site in a restricted area situated at the interface of subdomain1 and subdomain 2 in the vicinity of the NTS-DBL1α_1_ hinge region. This differs from the CSA-binding site localized on VAR2CSA DBL3X domain, which lies within subdomain 3 [47]–[49], and is more in line with the location reported for *P. knowlesi* Duffy Binding Protein (also a site engaging residues from subdomains 1 and 2) [50] or some of the sialic acid-binding sites of *P. falciparum* EBA175 [51]. The NTS-DBL1α_1_ hinge region, missing in our previous single domain structure, is highly exposed on the surface and proved to be crucial for the RBC-binding site. Indeed, cleavage of this sequence disrupted binding and mutations of this region reduced binding, without substantially affecting antigenicity (recognition of all mutants by ELISA was essentially unimpaired, data not shown). This reinforces the conclusion that NTS is an essential functional and structural component of the DBL1α domain [37]. Importantly, the blood group A binding site and the major heparin-binding site that we mapped previously [37]are distant from each other on the surface, indeed on opposite sides of the molecule. Therefore, direct competition with binding to the receptor cannot be the reason why heparin disrupts rosettes and inhibits the binding of the recombinant domains to RBC and trisaccharide-BSA conjugates, contrary to one of the previously suggested hypotheses [27]. The other possible mechanism, namely that heparin could provoke the formation of oligomers that are no longer competent for receptor binding, remains an interesting possibility. The RBC-binding site could become inaccessible in heparin-aggregated adhesins, or several PfEMP1 molecules need to bind simultaneously to the ABO antigens displayed on the RBC surface for an efficient interaction to occur and this is prevented in the heparin complexes.

**Figure 10:**
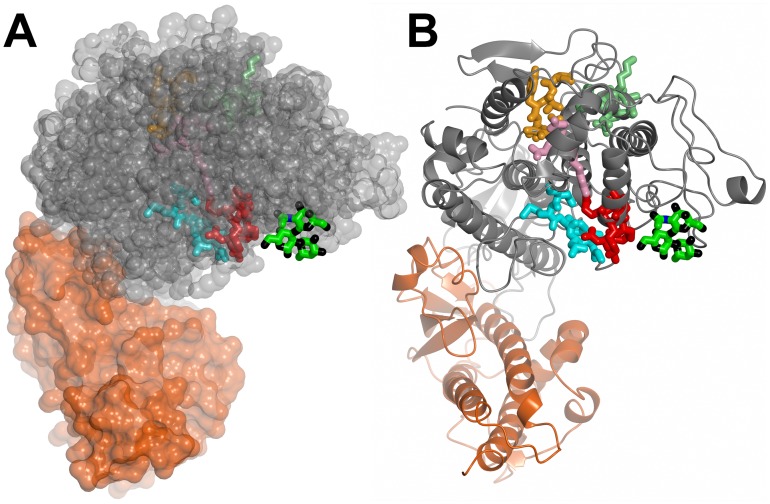
Localisation of the PolV1-4 and High rosetting motif H3 relative to the RBC binding site on DBL1α_1_. The PolV tags are shown in stick representation: PolV1 (sequence DMFLP, cyan), PolV2 (sequence LREDW, pink), PolV3 (sequence IWKALT, pale green) and PolV4 (sequence PTYLD, orange). Motif H3 is shown in red. The docked trisaccharide is shown as in Figure 8. (**A**) Space-filling representation and (**B**) ribbon representation. PolV2 and 3 lie in αH5, PolV4 motif is the main constituent of αH5′. PolV1 tag is located between αH3 and αH4, in the area adjacent to the binding site. All PolV are part of the scaffold and not accessible to the surface. Palo Alto 89F5 VarO has a H3 motif located at the beginning of αH4 (shown in red), which is surface exposed and located adjacent to the trisaccharide binding site. Motif H2 (TCA/GAK/TV/M) is located at the end of αH5 and is not well conserved in VarO (its sequence is TC_270_ SAPY). It is situated on the opposite face with respect to motif H3 and the RBC binding site (not shown). Motif H1 (RFSKN) is not present in VarO.

We analysed the location of the RBC-binding site on the DBL1α_1_ domain with respect to the position of molecular signature tags used to classify *var* genes and associate them with either severe or uncomplicated malaria. Conserved tags, called positions of limited variability 1 to 4 (PolV1-4), had been identified [52], [53]. The relative combination of PoLV motifs appears characteristic of specific *var* gene subsets. Figures 6 and 10 show the localization of the four PolV sequence tags with respect to the identified RBC binding site of PfEMP1-VarO. All PolV tags, except for PolV1, are remote from the RBC-binding site (Figure 10). Normark et al. [54] identified specific PfEMP1-DBL1αamino acid motifs correlated with rosetting and severe malaria.One of the sequence signatures associated with “high rosetting”, namely H3, maps close to the binding site identified here. Palo Alto 89F5 VarO has a H3 motif (H3 K D K/A V E/Q K G) located at the beginning of αH4, which includes K216, a residue critical for RBC binding (Figures 6 and 10). This motif is surface-exposed and located in close proximity of the RBC-binding site.

The RBC surface displays several million copies of ABO blood group determinants carried on membrane glycoproteins and glycolipids. The ABH antigens lie on terminal branches of poly-N-acetylgalactosamines, each of which may carry several ABH determinants. Although the type of branching varies, the ABH determinants displayed on the RBC surface are very dense. It is possible that PfEMP1 binding involves interaction with more than one glycan per Head region.Furthermore, although both DBL1α_1_ domain and the Head region are monomeric in solution, we do not know whether binding is associated with oligomerization of the adhesion domain, as reported for other RBC-binding proteins with DBL domains such as *P. falciparum* EBA175 [51] and the *P. vivax* Duffy Binding Protein [55].

The DBL1α_1_-CIDR1γ Head is present in a small subset of *var* genes from group A, four of which are implicated in rosetting [23], [25], [26]. The RBC-binding site is conserved in other rosette-forming PfEMP1 variants such as R29, PF13_003 and IT-var60, indicating that data obtained here can be extrapolated to other lines and form the molecular basis of the extensively documented ABO blood group preference in rosetting [9], [14]–[16]. The presence of CIDR1γ increases binding efficiency, as indicated by the approximately 1 log unit higher MFI in flow cytometry and the increased amount of protein bound, as visualised by immunoblotting. The exact role played by CIDR1γ, however, is still unclear. It is possible that its folding back upon the DBL1α_1_ domain provides a structural framework for more efficient binding and increased affinity, just as the multimodular PfEMP1-VAR2CSA has been shown to require a compact fold for activity [56], [57]. The binding characteristics of the Head region resemble those of the infected red cells, except for the susbtantial residual binding in the absence of human serum and the similar enhancement by human and foetal calf serum. Serum enhancement of NTS-DBL1α_1_ andHead binding to RBCs may reflect a need to buffer the highly negatively charged RBC surface. As the serum component(s) implicated in VarO rosetting and Head region binding are unknown, we carried out all binding assays in the presence of human serum. VarO rosetting has an absolute requirement for human serum (Figure S1A) that cannot be replaced by foetal calf serum. As we show that binding of NTS-DBL1α_1_ and/orHead does not account for this human-specific serum dependency, we suppose that interaction of serum components with downstream PfEMP1-VarO domains might contribute to modulate binding of PfEMP1-VarO, possibly by increasing affinity and optimising binding characteristics, or that other RBC surface proteins (eg. rifins or stevors) come into play in rosetting as well. Further work is needed to clarify this question.

This work provides the molecular basis underpinning the blood group preference of rosetting. The association between the ABO groups, rosetting and severe malaria [9] is a strong indication that rosetting, as a contributor to severe malaria, has exerted a selective pressure that has shaped population polymorphisms at the ABO locus and has contributed to their varying geographic distribution. The data reported here expand this framework to subgroups within the susceptible blood group A. Although the genetic basis of the A_1_, A_2_ and other rare A subgroups is well established, the physiological consequences of such phenotypes and the selective advantage they provide are unclear. The lower prevalence of A_1_ blood group in populations of African descent compared to populations of Asian or Caucasian origin [58]–[60] is consistent with the hypothesis that *P. falciparum* rosetting has contributed to subgroup selection and gene spread of blood group A variants.

## Materials and Methods

### Blood samples

For ABO groups, blood donated by healthy volunteers was purchased from the Blood Bank Centre (EFS, Rungis). Fresh or cryo-preserved and thawed RBCs of A_1_, A_2_ or A_x_ subgroup were obtained from the reference cryobank reagents of the Centre National de Référence pour les Groupes Sanguins (CNRGS - INTS, Paris). Blood samples with CR1 copy number variation were left-overs (“fond de tubes”) from healthy volunteers recruited by the URCA EA3798 (Reims, France) for clinical studies. Specific written consent was provided by each donor to use the left-overs for research. The Comité Consultatif pour la Protection des Personnes se prêtant à des Recherches Biomédicales of Champagne Ardenne approved the protocol. Supply and handling of human red cells followed the guidelines of the agreement between Institut Pasteur and the Etablissement Français du Sang.

### Determination of CR1, A and H antigen levels on RBCs

The quantification of CR1 copy number was assessed using biotinylated anti-CR1/CD35 mAb J3D3, followed by a sequential labelling with streptavidin-phycoerythrin (PE), biotinylated anti-streptavidin and streptavidin-PEantibodies as described [61], [62].The amount of A and H antigens displayed on RBC surface was determined by flow cytometry using the following monoclonal antibodies: mAb BRIC-145/9W2 (mouse anti-A antigen, IgG1) and mAb MR3-517 (mouse anti-H antigen, IgM), respectively, and confirmed using TransClone Anti-ABO1 (IgM) and TransClone anti-H1 (IgM) murine mAbs (Bio-Rad Laboratories), respectively. Secondary goat anti-mouse IgG or IgM Alexa fluor 488-conjugated (Molecular Probes, Invitrogen) antibodies were subsequently used. To reduce agglutination of antigen-positive cells, RBC samples were fixed for 10 min at room temperature with 0.1% glutaraldehyde as described [61] before staining. For each sample, 50,000 events were collected using a BD-LSR1 flow cytometer (Becton Dickinson) and expression levels were analysed with the FlowJo 9.4.7 software.

### Parasite cultures and rosetting characteristics

Monovariant cultures of the Palo Alto 89F5 VarO and It4/R29 parasites, procedures for rosette enrichment on ice-cold Ficoll, rosette dissociation with dextran sulphate, magnetic selection of mature iRBCs were as described [24], [25]. Rosette reformation assays were carried out in RPMI with 10% AB human serum (RPMI-HS) 1 h at 37°C and the rosetting rate was calculated, after addition of Hoechst 33342 dye (Molecular Probes) for parasite nuclei staining, by determining the percentageof rosette-forming iRBCs present in the mature parasite population.

For experiments exploring the parameters affecting rosetting, purified VarO-iRBCs were diluted in RPMI-HS in the presence of specific mouse mAbs or isotype controls, or diluted in a range of human serum concentrations or 10% Ig-depleted human serum. Ig depletion of the serum was achieved by incubating 3 mL of RPMI-HS with 1 mL of RMPI-equilibrated protein G Sepharose beads (GE Healthcare). Depletion was assessed by Coomassie blue staining (PageBlue Protein Staining Solution, Thermo Scientific) of sodium dodecyl sulphate-polyacrylamide gel electrophoresis (SDS-PAGE) and immunoblotting using anti-human Ig. Enzyme treatment of recipient RBCs was carried out by incubating 5×10^7^ RBCs (washed twice in PBS, Phosphate Buffer Saline 1×, Invitrogen) for 30 min at 37°C with 10 µg trypsin (Sigma, T1005) or α-chymotrypsin (Sigma, C4129) in a final volume of 100 µL. Enzymatic digestion was stopped by three extensive washes in RPMI-HS. Surface expression of CR1/CD35 was detected using anti-CR1 mAb J3B11, followed by an Alexa fluor 488-conjugated goat anti-mouse IgG. Treated or untreated recipient RBCs were used in rosette reformation assays with magnetically enriched mature VarO-iRBCs.

For ABO blood group preference assays, red cell membranes were labelled according to the manufacturer's instructions with the lipophilic fluorescent probes PKH67 or PKH26 (Sigma Aldrich), both of which provide stable, clear, intense and reproducible fluorescent labelling of live red cells with no apparent loss of function [62] or antigenicity [25]. VarO-iRBCs were purified to >90% parasitemia by magnetic separation and diluted in the presence of varying ratios of recipient A, B and O RBCs differentiated by labelling alternately with PKH26 or PKH67. After incubation in RPMI-HS for 1 hr at 37°C, the rosetting rate was evaluated by fluorescence microscopy.

### Cloning, expression and purification of recombinant domains

The Palo Alto VarO coding sequence (GenBank EU9082205) was used to design synthetic genes with a recodoned sequence to restore a balanced A+T/G+C ratio with all predicted N-glycosylation NxT/S sites mutated to NxA, except for DBL1α_1_(wt) and Head(wt). DBL1α_1_ (residues 2–471) and DBL1-long (residues 2–487) were cloned in pMAL-p2X (NewEngland Biolabs) between the NdeI-NotI restriction sites. DBL1α_1_(wt) (residues 1–471), Head (residues 2–716) and Head(wt) (residues 1–716) were cloned in pMAL-c2X (NewEngland Biolabs) between the BamHI-HindIII restriction sites. CIDR1γ (residues 508–787) was cloned in pET15 (Novagen) between the NdeI-XhoI restriction sites. DBL2β (residues 831–1206) and DBL3γ (residues 1220–1578) were cloned in pET22b (Novagen) between the NdeI-HindIIIrestriction sites.DBL4ε (residues 1608–2014) and DBL5ε (residues 2025–2321) were cloned in pMAL-c2X between the EcoRI-SalI restriction sites. All constructs, apart from DBL1α_1_ and Head had an in-frame hexa-His tag at the C-terminus (N-terminus in the case of CIDR1γ). A thrombin cleavage site was introduced by PCR before the hexa-His tag in the DBL2β, DBL4ε and DBL5ε constructs. The recombinant domains were expressed in Rosetta-Gami (Novagen) except for DBL3γ, DBL1α_1_(wt) and Head(wt), which were expressed in SHuffle (New England Biolabs).

Protein expression in *Escherichia coli* was carried out for 20 h at 20°C (16°C for the Head) and purified by affinitychromatography (TALON, Clontech), followed by size exclusionchromatography (S200 or S75 16/60, GeHealthcare) as described [37]. In the case of MBP fusion proteins, MBP was cleavedusing Factor Xa (Novagen) and the recombinant domain purified as described [37]. Where necessary, the hexa-His tag was cleaved by thrombin (Novagen) as recommended by the manufacturer and the cleavage monitored by Western blot. For crystallisation, the Head protein was further purified on a HiTrap Heparin column (GE Healthcare), followed by size exclusion chromatography(S20016/60, GE Healthcare) in 20 mM Tris-HCl, 200 mM NaCl, pH8.0 and on a CM ion-exchange column with an NaCl gradient from 50 mM to 1 M NaCl. The protein in a final buffer (20 mM Tris-HCl, pH 8, 200 mM NaCl) was then concentrated to 10 mg.mL^−1^.

The PvDBP construct used as control for some experiments encompassed residues 199–515 of the *P. vivax* Duffy Binding protein. The domain was cloned in baculovirus, produced in HiFive cells, and purified as described [24].

### Mutagenesis

Point mutations were made using polymerase chain reaction-based mutagenesis kits (Quikchange Lightning Site-Directed Mutagenesis kit or Quikchange Multi Site-Directed Mutagenesis kit, Stratagene) following the supplier protocols. Mutants Mut1-7 have been described previously [27]. Mutants 12 and 13 were custom made by GeneCust (Luxembourg) using DBL1α_1_ as template. Mutants 10 and 14–19 were custom-made by GenScript (Hong Kong) using DBL1α_1_ and DBL1α_1_(wt) as template, respectively. All mutants were produced as MBP fusion proteins and purified as above.

### Immune reagents and assays

Antibodies to the individual PfEMP1 domains and the Head were produced by immunising OF1 mice, five mice per antigen, (6–8 weeks old mice, Charles River, France) with 10 µg recombinant protein in Freund's Complete Adjuvant for the first injection and Freund's Incomplete Adjuvant for subsequent injections done at days 21 and 42. Serum was recovered ten days after the third injection and stored at −20°C until used. Antibody titers were determined by ELISA on the cognate antigen [24]. Immunoblot reactivity with PfEMP1 was assessed on parasite extracts separated on 4–12% gradient SDS-PAGE (Biorad) under reducing and non-reducing conditions. Reactivity with the VarO-iRBC surface was assessed by fluorescence microscopy using a Leica DM5000B Fluorescence microscope [24] and flow cytometry using a BD-LSR1 cytometer [25]. Monoclonal anti-CIDR1γ antibody, mAb G8-49, was isolated from an OF1 mouse (as described inNato et al. [63]) immunised with the Head protein by screening by ELISA and VarO-iRBC surface reactivity. Monoclonal IgG was precipitated with 50% saturated ammonium sulphate from ascitic fluid, centrifuged, and dialyzed against PBS.

### Binding of recombinant PfEMP1-VarO proteins to RBCs

For each binding assay, 2.5×10^−11^ mole protein was incubated at RT for 30 min with 2×10^7^ RBCs in 100 µL RPMI-HS. RBCs were separated from the incubation mixture by centrifugation through 200 µL 85% silicone DC550 (Serva) 15% Nujol (Alfa Aesar) as described [27]. After one wash in RPMI, RBCs were either processed for immunoblotting [37]or for flow cytometry analyses by incubating with a specific mouse polyclonal serum followed by Alexa fluor-488-conjugated goat anti-mouse IgG F(ab′)_2_(Molecular probes) [25]. For flow cytometry analysis, the Head region was pre-incubated with 50 µg mAb G8.49 before addition of RBCs in the assay. 50,000 events were recorded using LSR1 flow-cytometer (Becton Dickinson) and analysed with FlowJo software.

### Surface plasmon resonance assays

Assays were performed on a Biacore2000 instrument (GE Healthcare) in PBS buffer at 25°C. Trisaccharide A or B-conjugated BSA (6 atom spacer, average of 19 and 21 sugar residues per protein molecule for group A or B, respectively; Dextra Laboratories Ltd, UK) and unconjugated BSA (Sigma-Aldrich) were covalently coupled to the linear polycarboxylate hydrogel surface of three independent flowcells of a HLC 200 m sensorchip (Xantec bioanalytics), using the Amine coupling kit (GE Healthcare). Immobilization densities of 2500 resonance units (RU; 1RU = 1 pg.mm^−2^) were attained. A duplicate range of concentrations (10 nM–1 µM) with or without a 2-fold excess of heparin (heparin 5000; Sigma-Aldrich) of Head(wt) was then flowed at 50 µL.min^−1^ over the three surfaces. The association and dissociation profiles were double referenced using the Scrubber 2.0 software (BioLogic Software), i.e. both the signals from the unconjugated BSA reference surface and from blank experiments using interaction buffer instead of protein were subtracted. The shape of the profiles was suggestive of a complex binding mechanism that was not explored further in the context of this study.

### Crystallisation and structure determination

The protein (10 mg.mL^−1^ final concentration) was mixed with heparin 5000 (Sigma-Aldrich) at a slight molar excess. Crystals were grown using the hanging-drop vapour-diffusion method: 1 µL of protein solution was mixed with 1 µL of reservoir solution (10% PEG 3350, 200 mM NaCl, 100 mM sodium citrate, pH 8.3) and equilibrated against 0.5 mL of reservoir solution. Crystals appeared after 6–8 hours and continued to grow for about one week. The crystals were passed through a cryoprotectant solution (20% glycerol, 15% PEG 3350, 200 mM NaCl, 100 mM sodium citrate, pH 8.3) and frozen in liquid nitrogen.

Data were collected on the beamline PROXIMA1 at SOLEIL (St. Aubin, France). All data were treated with XDS [64], followed by SCALA from the CCP4 program suite [65]. The structure was solved by molecular replacement using the high-resolution structure of the DBL1α_1_ domain [37]Protein Data Bank (PDB entry 2xu0) as a search model. Several cycles of refinement using Buster [66] and rebuilding the structure using Coot [67]allowed to identify density belonging to the CIDR1γ domain. This was fitted using Buccaneer from the CCP4 programme suite and further refined with Buster. The conserved three-helix motif from the CIDRα domain of the MC strain [28] was used to search for a similar motif in the electron density map to allow matching the sequence of the protein and to build the remaining structure. The refined coordinates and structure factors have been deposited in the Protein Data Bank (PDB entry code 2yk0). Structural figures were prepared withCCP4MG [68].

### Computer docking

Computer docking of trisaccharides onto the Head structure was performed using Autodock version 4.2 [69]. Coordinates for trisaccharide A were taken from a complex in the Protein Data Bank (PDB entry code 2obs). The search was performed over the surface of the region of the DBL1α_1_ moiety encompassing the NTS segment, subdomains 1 and 2, in order to allow fine grid sampling (0.375 Å). Default values were usedfor all docking parameters, except the number of search runs, which was 100. The 10 best solutions were retained.

### Analytical ultracentrifugation

The protein samples (0.5–2 mg.mL^−1^) were analysed in a Beckman Coulter XL-I analytical ultracentrifuge. Detection of the protein concentration as a function of radial position and time was performed by optical density measurements at a wavelength of 280 nm or 250 nm for high concentration samples. All samples were in a 50 mM NaCl and 20 mM Tris pH8 buffer. All experiments were carried out in an An-Ti 50 rotor at 20°C at a rotor speed of 42,000 rpm for 8 hours. Sedimentation velocity analysis was performed by continuous size distribution analysis c(s) using Sedfit 12.0 [70]. Partial specific volume 0.726 mL.g^−1^ and 0.738 mL.g^−1^ for the CIDR1γ and DBL1α_1_-CIDR1γ respectively, viscosity 0.01013 Poise and density 1.00093 g.mL^−1^ were calculated using Sednterp 1.09 and used to analyze experimental data. Sedimentation coefficients at zero concentration were obtained by linear extrapolation to zero concentration of the sedimentation measured for each protein sample at different concentrations. Sedimentation coefficients were corrected for viscosity and expressed as values in water at 20°C. Theoretical sedimentation values were calculated with the programmes HYDROPRO 7c [41] and US-Somo [40].

### Circular Dichroism (Far UV)

Far-UV CD spectra were measured (Aviv215 spectropolarimeter, Aviv Biomedical)using a cylindrical cell with a 0.01 cm path length as described [37]. The spectra, corrected using buffer baselines measured under the same conditions, were normalised to the molar peptide bond concentration and path length as mean molar differential coefficient per residue.

### Ethics statement

Blood donation in France includes an optional consent for use of part or all of the donation for teaching, research purposes or for preparation of specific reagents - (see documenthttp://www.dondusang.net/content/medias/media1837_FsPssqwYyfXZQcm.pdf?finalFileName=Document_dinformation_pr-don.pdf). All donors participating to this study had given their written consent for this use. Healthy volunteers donating RBCs with known CR1 copy number were recruited by the URCA EA3798 in Reims as part of an ongoing clinical study on CR1. The samples used here were left-overs (“fond de tubes”), for which specific written consent was provided by each donor for use in research on other diseases. The Comité Consultatif pour la Protection des Personnes se prêtant à des Recherches Biomédicales Est II approved the protocol (Ref protocol CCP:11/603, Ref Afssaps 2011-A00594-37). Supply and handling of human red cells followed the guidelines of the agreement between Institut Pasteur and the Etablissement Français du Sang and the regulation of blood donation in France. The IMP Unit at IP was issued an Habilitation à manipuler du sang humain (HS2003-3255; ref ND/LK/CC-11.68).

For animal use, the study was carried out in strict accordance with the recommendations in the Guide for the Care and Use of Laboratory Animals of the Institut Pasteur and complied with the European Union guidelines for the handling of laboratory animals (http://ec.europa.eu/environment/chemicals/lab_animals/home_en.htm). The procedures were approved by the Institut Pasteur animal care and use committee. Animal care and handling was approved by the Ministère de l'Agriculture et de la Pêche (rapport 107503056792, clearance number 75–273, issued to OMP) and the protocols and procedures used by the Direction Départementale des Services Vétérinaires de Paris (Ref. RL- 07031395-30701147, issued to OMP). All animal experiments were planned in order to minimize mice suffering.

### Statistical analysis

Differences in mean fluorescence binding of DBL1α_1_(wt) or Head(wt) between ABO RBCs or A subgroup RBCs were tested using the non-parametric Kruskall-Wallis test. The associations between recombinant protein binding and blood group A or H antigens expression levels found to be significant using the Spearman's correlation analysis were investigated by linear regression analysis. The statistical analyses were done with STATA software (STATA Corp. Release 9.0). For all tests, P values of less than 0.05 were considered statistically significant.

### List of accession numbers/ID numbers for genes and proteins

VarO cDNA sequence: GenBank database accession number EU908205

High-resolution structure of the DBLα_1_ domain: Protein Data Bank entry 2xu0

Refined coordinates and structure factors of Head: Protein Data Bank entry 2yk0

## Supporting Information

Figure S1 **Rosetting characteristics of the monovariant Palo Alto 89F5 VarO parasite culture.** (**A**) VarO Rosette formation is human serum dependent. VarO rosettes were prepared from monovariant cultures [24], dissociated with dextran sulphate and washed twice in RPMI without human serum. Rosette reformation assays were performed in RPMI medium in the presence of increasing concentration of AB human serum (1 to 10%). Rosetting rate was counted by microscopic examination after incubation at 37°C for 1 hour. Results of three independent assays. (**B**) VarO rosetting is IgG- and IgM-independent. Non-immune human serum was depleted from Ig by protein-G Sepharose chromatography. Rosette reformation assays were performed as in (**A**). Results show the rosetting rate of VarO iRBCs cultivated under standard conditions (1) and VarO rosettes formed in unfractionated serum (2) or Ig-depleted serum (3). Results are from three independent assays. (**C**) VarO rosettes do not bind non-immune IgG or IgM. VarO rosettes cultivated in RPMI-10% non-immune AB human serum (NIS) were resuspended in PBS supplemented with 2% fetal calf serum and incubated with goat anti-human IgG (Molecular Probes, A11013) or IgM (Molecular Probes, A21215) Alexa fluor 488 conjugated antibodies. Hoechst dye was added in each sample to detect iRBCs (FL4 fluorescence). After an incubation of 30 min at 37°C, samples were washed twice in PBS-FCS and surface immuno-staining was analysed by flow cytometry. Representative results (of at least three independent experiments) including the background labelling (no antibodies added) and a positive control labelling obtained after incubation of the rosette enriched samples with a pool of hyper-immune sera (HIS) collected from Senegalese adults [24] are shown.
(TIF)

Figure S2 **PfEMP1-varO derived recombinant domains.** (**A**) PfEMP1-varO domain architecture and schematic representation of the recombinant domains. DBL: Duffy Binding Like domain; CIDR: Cysteine-rich Inter Domain Region; TM: Trans-Membrane region; ATS: Acidic Terminal Segment (also known as VarC). The boundaries of each domain (amino acid residues) and the predicted molecular mass are indicated. Note that for DBL1α_1_ and the Head, the wt constructs started at residue 1 while the constructs with mutated N-glycosylation sites started at residue 2. All domains apart from DBL1α_1_(wt)and Head(wt) had all potential N-glycosylation NxT/S sites [number of sites indicated on the left in italics] mutated to NxA. (**B**) SDS-PAGE analysis of the recombinant domains (2 µg/lane) run in reducing conditions and stained with Coomassie BlueR (Biorad): (lane 1) DBL1α_1_ (18 Cys, no hexa-His tag), (lane 2) DBL1α_1_(wt) (18 Cys, hexa-His tag), (lane 3) DBL1α_1_-long (20 Cys, no hexa-His tag), (lane 4)Head (no hexa-His tag), (lane 5) Head(wt) (hexa-His tag), (lane 6) CIDR1γ, (lane 7) DBL2β, (lane 8) DBL3γ, (lane 9) DBL4ε, (lane 10) DBL5ε. (**C**) Far UVcircular dichroism spectra (CD) of the various recombinant domains [37]. The recombinant domains are colour-coded as indicated. The table on the right presents the secondary structure estimations derived from the normalized spectra using the CDSSTR method included in the CDPro software [72].
(TIF)

Figure S3 **mAb G8.49 reacts with CIDR1γ-VarO.** Specificity of mAb G8.49, isolated from an OF1 mouse immunised with the Head domain, was tested by ELISA on plates coated with 1 µg/mL (100 ng/well) of DBL1α_1_, DBL1α_1_(wt), CIDR1γ, Head and Head(wt).
(TIF)

Figure S4 **Seroreactivity of the DBL1α_1_ and Head proteins is unaffected by mutation of the predicted N-glycosylation sites.** Titration of polyclonal mouse sera raised to the recombinant DBL1α_1_ domain (**A**) or to the recombinant Head domain (**B**) on the DBL1α_1_, DBL1α_1_(wt), Head and Head(wt) domains.
(TIF)

Figure S5 **Factors influencing binding of the DBL1α_1_(wt) and the Head(wt).** (**A**) **&** (**B**) Binding of DBL1α_1_(wt) and the Head(wt) to RBC is inhibited by heparin and by mAb D15-50. Histogram representation of flow cytometry analysis of protein bound to blood group A_1_ RBCs. Assays for RBC binding to DBL1α_1_(wt) and the Head(wt) were conducted in RPMI supplemented with 10% human serum in the presence or absence of 1 mg.mL^−1^ heparin (which disrupts VarO rosettes) [37](**A**) or after pre-incubation of the protein with 50 µg of the VarO rosette-disrupting mAb D15-50 [24](**B**). Residual protein binding was assessed by flow cytometry. (**C**) **&** (**D**)**.** RBC binding to DBL1α_1_(wt) and the Head(wt) domains is potentiated by serum. Frequency histogram of flow cytometry analysis of protein bound to blood group A_1_ RBCs in the absence of human serum or in the presence of 10% AB human serum (green) or 10% foetal calf serum (blue) (**C**). Immunoblot analysis of the bound protein of the samples incubated in the presence (+) or absence (−) of 10% human AB^+^ serum, using mouse polyclonal antibodies to DBL1α_1_, revealed using alkaline phosphatase (**D**). The right panel shows an immunoblot analysis of binding of PvDBP (38 kDa), which is serum-independent. Scanning of the immunoblot (lower panel) concurred with the results of flow cytometry.
(TIF)

Figure S6 **Relationship between RBC binding to DBL1α_1_(wt)and the Head(wt) and amount of A or H antigen displayed on the RBC.** Graphs represented the linear regression plots of the association between the binding level [Mean Fluorescence Intensity (MFI) recorded by flow cytometry] of DBL1α_1_(wt) or Head(wt) and blood group A or H antigen expression levels. (**A**) Association between binding levels on A RBC subgroups (A_1_, A_2_ and A_x_) and blood group A antigen expression. (**B**) Association between binding levels on A_1_, A_2_, A_x_, B and O RBC and H antigen expression. For each graph, the regression equation and the coefficient of determination (R^2^) are shown.
(TIF)

Figure S7 **Binding of Head(wt) to BSA-conjugated A or B trisaccharide.** Real-time association and dissociation profiles monitored by surface plasmon resonance, corresponding to the injection of different concentrations of the Head(wt)domain, over immobilized BSA-conjugated trisaccharide A (left) or trisaccharide B (right).
(TIF)

Figure S8 **Recombinant DBL1α_1_ domains cleaved after R64 or R69 no longer bind RBC.** The left panel shows a Coomassie Blue R-stained SDS-PAGE gel, with evidence of cleavage of DBL1α_1_(lane 1), which migrated as two bands. N-terminal sequencing showed that the lower band (dotted arrow) started at residue N_70_SDG (i.e. the protein was cleaved after R69). Lane 2 shows a factor Xa-cleaved Mut10, in which an internal factor Xa cleavage has been inserted at positions 66–69 (R_64_YGYVR_69_ mutated to R_64_YIEGR_69_), and the domain released from its MBP carrier by thrombin cleavage (the plasmid was engineered to create a thrombin cleavage site after the MBP coding sequence). The proteins were used in RBC binding assays and binding was visualised by immunoblot to identify the RBC-bound band(s) (right panel), DBL1α_1_ (lane 1), cleaved Mut10 (lane 2) or flow cytometry - DBL1α_1_(panel 1), cleaved Mut10 (panel 2).
(TIF)

Figure S9 **Binding characteristics of the mutantdomains.** (**A**) Immunoblot of a typical RBC assay with Mut0 (lane 1), Mut1 (lane 2), Mut2 (lane 3), Mut3 (lane 4), Mut4 (lane 5), Mut5 (lane 6), Mut6 (lane 7), Mut7 (lane 8) and Mut10 (lane 9) probed with a polyclonal mouse anti-DBL1α_1_antiserum. M; molecular mass markers. (**B**) Dot plot representation of flow cytometry analysis of DBL1α_1_(wt) and Mut10-13 binding to A_1_ blood group RBCs. (**C**) Dot plot representation of flow cytometry analysis of Mut14-19 constructed on the DBL1α_1_(wt) background. Protein binding was probed with a polyclonal mouse anti-DBL1α_1_antiserum. The histogram on the right shows the distribution of fluorescence intensity for each mutant, colour-coded as indicated. Representative results of three independent assays.
(TIF)

Figure S10 **Superposition of CIDR1γ upon DBL1α_1_.** Superposition of the domains showing correspondence of helices (left) and β strands (right). Equivalent secondary structure elements are labelled (DBL1α_1_ followed by CIDR1γ). The domains are shown in ribbon representation with the subdomain colour code as in Figures 5 and 6.
(TIF)

Table S1 **Analysis of antibodies induced by the recombinant domains in outbred OF1 mice.**
(DOC)

Table S2 **CIDR1γ domain disulfide bonds.**
(DOC)

Table S3 **Sedimentation coefficient (S_20,w_) and frictional ratio (f/f_0_) of the recombinant domains.**
(DOC)
