# Supplementary figures and images for: Structural Basis for the ABO Blood-Group Dependence of Plasmodium falciparum Rosetting
Source: PLoS Pathog. 2012 Jul 12;8(7):e1002781. doi: 10.1371/journal.ppat.1002781 (PMC3395597; doi:10.1371/journal.ppat.1002781)

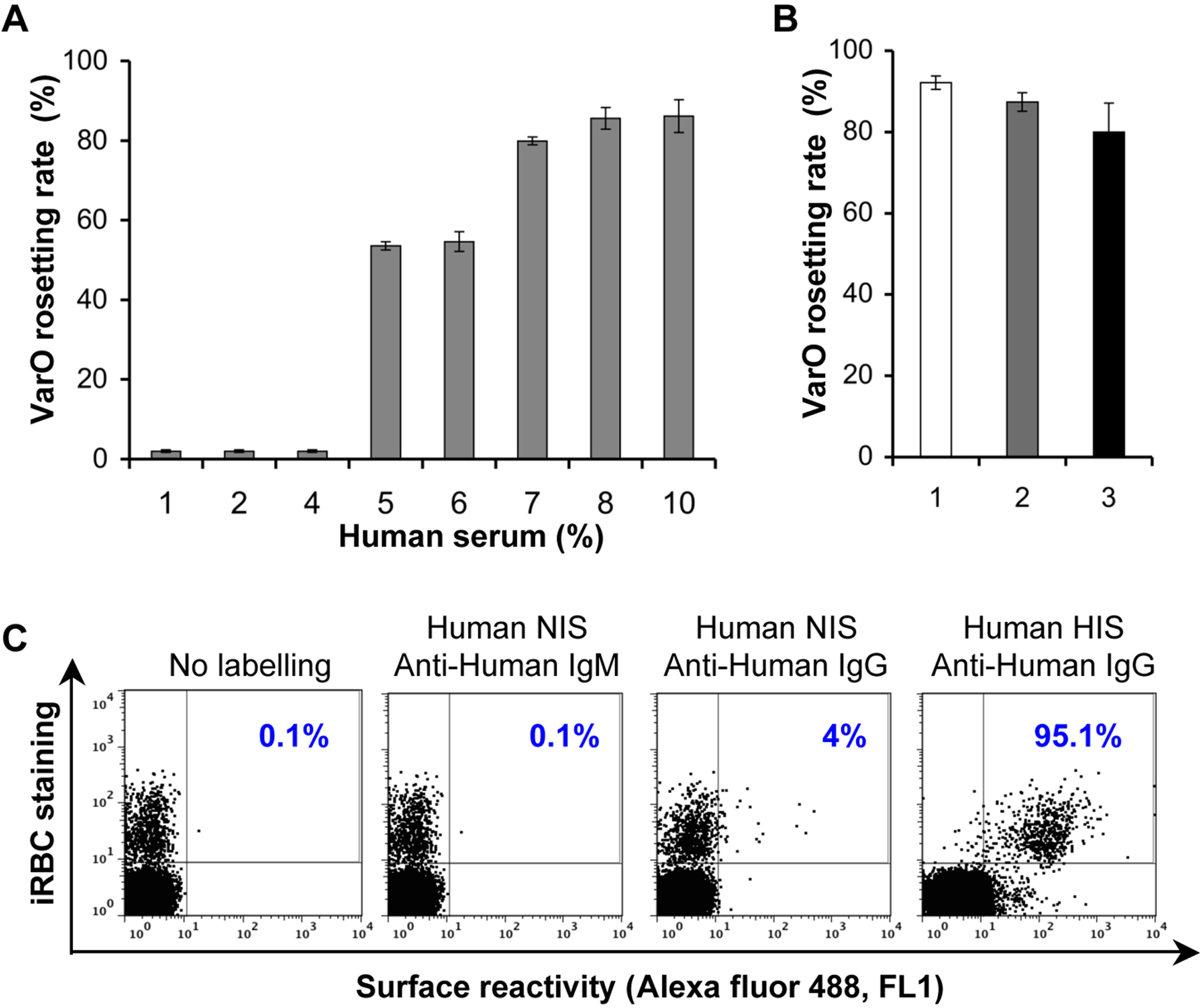

Supplement: Figure S1 — Rosetting characteristics of the monovariant Palo Alto 89F5 VarO parasite culture. (A) VarO Rosette formation is human serum dependent. VarO rosettes were prepared from monovariant cultures [24], dissociated with dextran sulphate and washed twice in RPMI without human serum. Rosette reformation assays were performed in RPMI medium in the presence of increasing concentration of AB human serum (1 to 10%). Rosetting rate was counted by microscopic examination after incubation at 37°C for 1 hour. Results of three independent assays. (B) VarO rosetting is IgG- and IgM-independent. Non-immune human serum was depleted from Ig by protein-G Sepharose chromatography. Rosette reformation assays were performed as in (A). Results show the rosetting rate of VarO iRBCs cultivated under standard conditions (1) and VarO rosettes formed in unfractionated serum (2) or Ig-depleted serum (3). Results are from three independent assays. (C) VarO rosettes do not bind non-immune IgG or IgM. VarO rosettes cultivated in RPMI-10% non-immune AB human serum (NIS) were resuspended in PBS supplemented with 2% fetal calf serum and incubated with goat anti-human IgG (Molecular Probes, A11013) or IgM (Molecular Probes, A21215) Alexa fluor 488 conjugated antibodies. Hoechst dye was added in each sample to detect iRBCs (FL4 fluorescence). After an incubation of 30 min at 37°C, samples were washed twice in PBS-FCS and surface immuno-staining was analysed by flow cytometry. Representative results (of at least three independent experiments) including the background labelling (no antibodies added) and a positive control labelling obtained after incubation of the rosette enriched samples with a pool of hyper-immune sera (HIS) collected from Senegalese adults [24] are shown. (TIF) [file ppat.1002781.s001.tif]

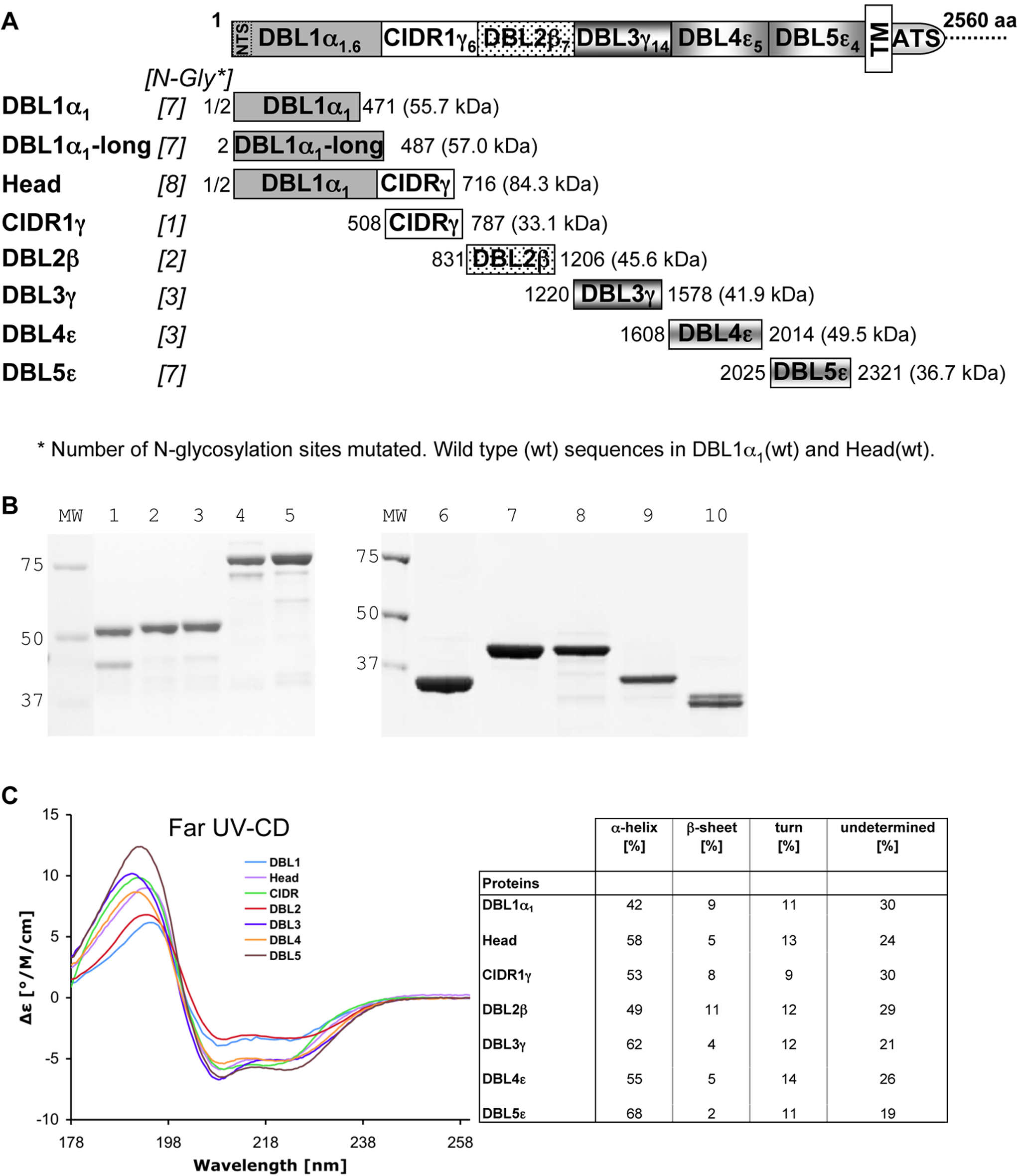

Supplement: Figure S2 — PfEMP1-varO derived recombinant domains. (A) PfEMP1-varO domain architecture and schematic representation of the recombinant domains. DBL: Duffy Binding Like domain; CIDR: Cysteine-rich Inter Domain Region; TM: Trans-Membrane region; ATS: Acidic Terminal Segment (also known as VarC). The boundaries of each domain (amino acid residues) and the predicted molecular mass are indicated. Note that for DBL1α1 and the Head, the wt constructs started at residue 1 while the constructs with mutated N-glycosylation sites started at residue 2. All domains apart from DBL1α1(wt)and Head(wt) had all potential N-glycosylation NxT/S sites [number of sites indicated on the left in italics] mutated to NxA. (B) SDS-PAGE analysis of the recombinant domains (2 µg/lane) run in reducing conditions and stained with Coomassie BlueR (Biorad): (lane 1) DBL1α1 (18 Cys, no hexa-His tag), (lane 2) DBL1α1(wt) (18 Cys, hexa-His tag), (lane 3) DBL1α1-long (20 Cys, no hexa-His tag), (lane 4)Head (no hexa-His tag), (lane 5) Head(wt) (hexa-His tag), (lane 6) CIDR1γ, (lane 7) DBL2β, (lane 8) DBL3γ, (lane 9) DBL4ε, (lane 10) DBL5ε. (C) Far UVcircular dichroism spectra (CD) of the various recombinant domains [37]. The recombinant domains are colour-coded as indicated. The table on the right presents the secondary structure estimations derived from the normalized spectra using the CDSSTR method included in the CDPro software [72]. (TIF) [file ppat.1002781.s002.tif]

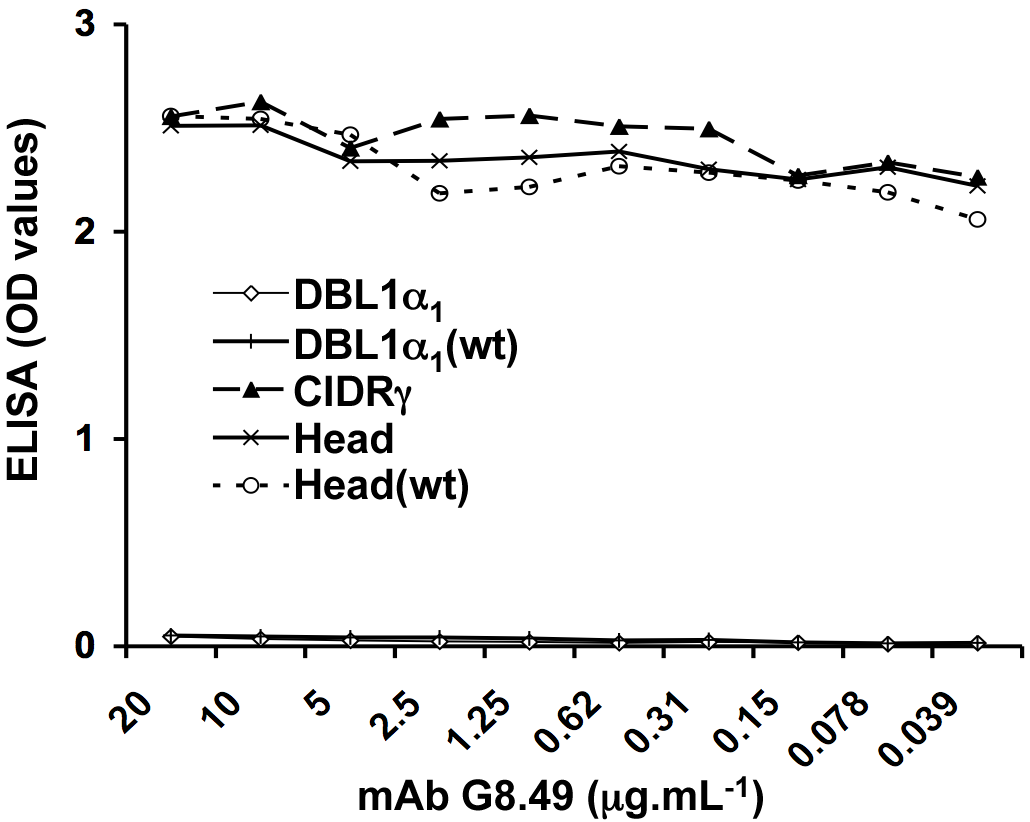

Supplement: Figure S3 — mAb G8.49 reacts with CIDR1γ-VarO. Specificity of mAb G8.49, isolated from an OF1 mouse immunised with the Head domain, was tested by ELISA on plates coated with 1 µg/mL (100 ng/well) of DBL1α1, DBL1α1(wt), CIDR1γ, Head and Head(wt). (TIF) [file ppat.1002781.s003.tif]

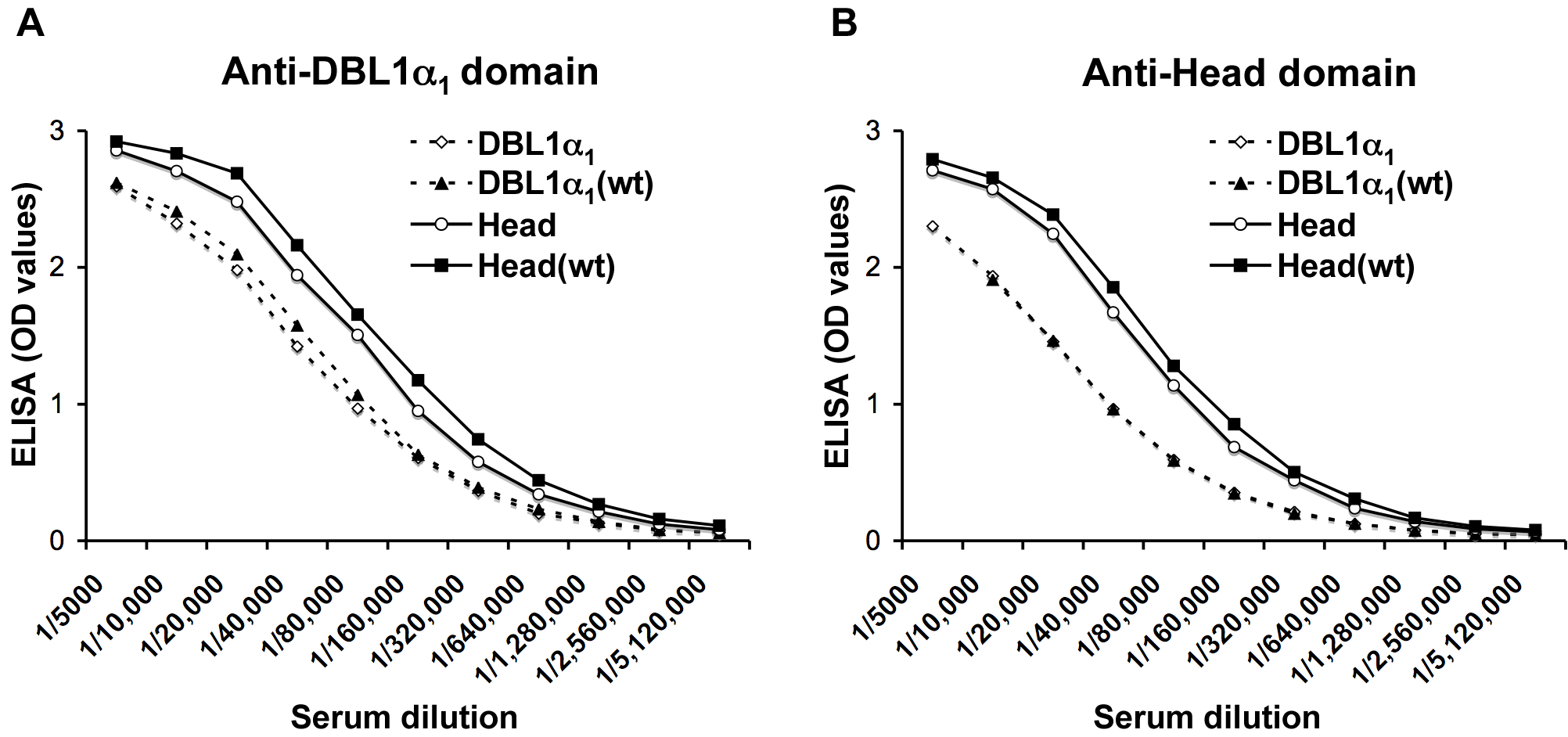

Supplement: Figure S4 — Seroreactivity of the DBL1α1 and Head proteins is unaffected by mutation of the predicted N-glycosylation sites. Titration of polyclonal mouse sera raised to the recombinant DBL1α1 domain (A) or to the recombinant Head domain (B) on the DBL1α1, DBL1α1(wt), Head and Head(wt) domains. (TIF) [file ppat.1002781.s004.tif]

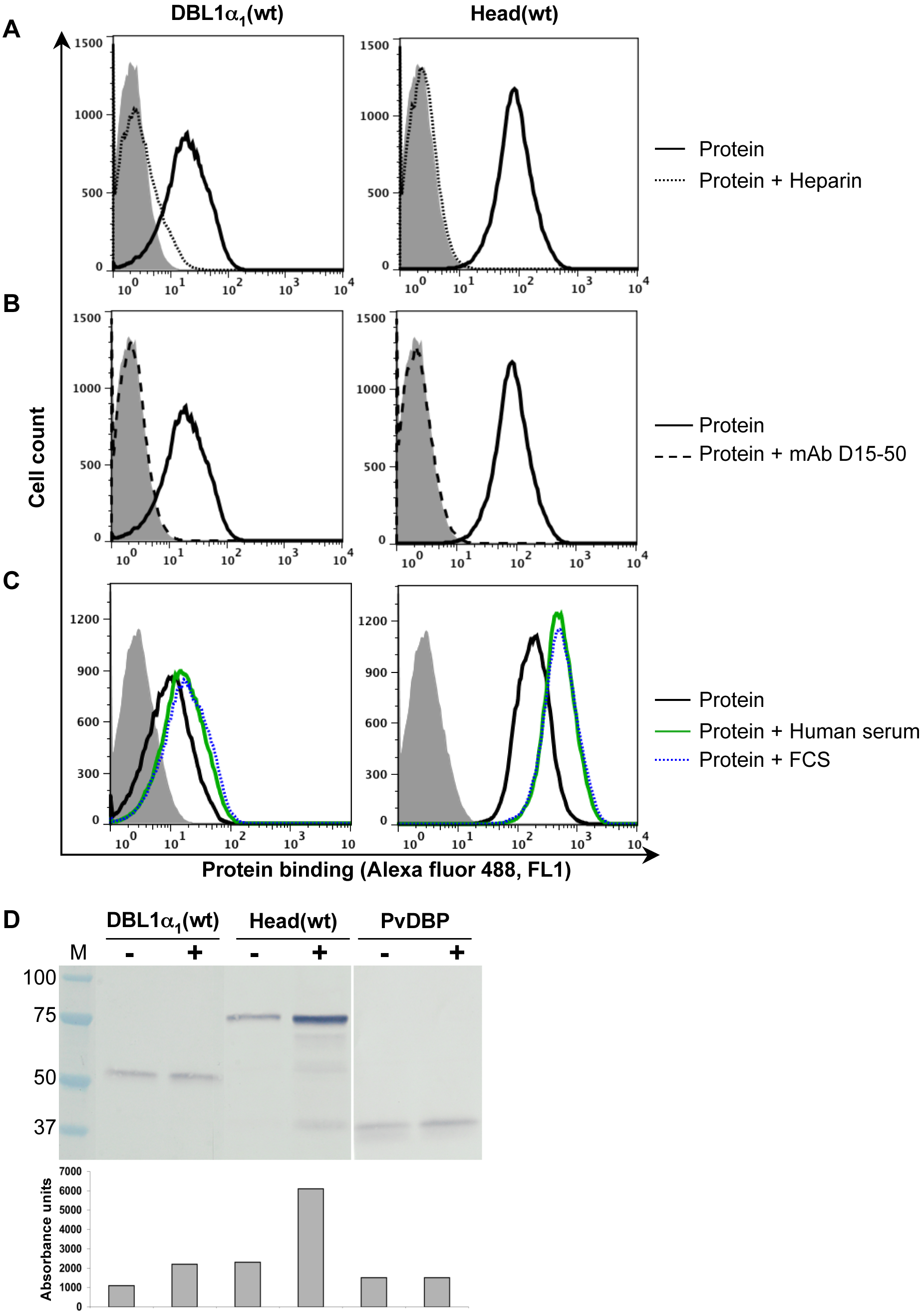

Supplement: Figure S5 — Factors influencing binding of the DBL1α1(wt) and the Head(wt). (A) & (B) Binding of DBL1α1(wt) and the Head(wt) to RBC is inhibited by heparin and by mAb D15-50. Histogram representation of flow cytometry analysis of protein bound to blood group A1 RBCs. Assays for RBC binding to DBL1α1(wt) and the Head(wt) were conducted in RPMI supplemented with 10% human serum in the presence or absence of 1 mg.mL−1 heparin (which disrupts VarO rosettes) [37](A) or after pre-incubation of the protein with 50 µg of the VarO rosette-disrupting mAb D15-50 [24](B). Residual protein binding was assessed by flow cytometry. (C) & (D). RBC binding to DBL1α1(wt) and the Head(wt) domains is potentiated by serum. Frequency histogram of flow cytometry analysis of protein bound to blood group A1 RBCs in the absence of human serum or in the presence of 10% AB human serum (green) or 10% foetal calf serum (blue) (C). Immunoblot analysis of the bound protein of the samples incubated in the presence (+) or absence (−) of 10% human AB+ serum, using mouse polyclonal antibodies to DBL1α1, revealed using alkaline phosphatase (D). The right panel shows an immunoblot analysis of binding of PvDBP (38 kDa), which is serum-independent. Scanning of the immunoblot (lower panel) concurred with the results of flow cytometry. (TIF) [file ppat.1002781.s005.tif]

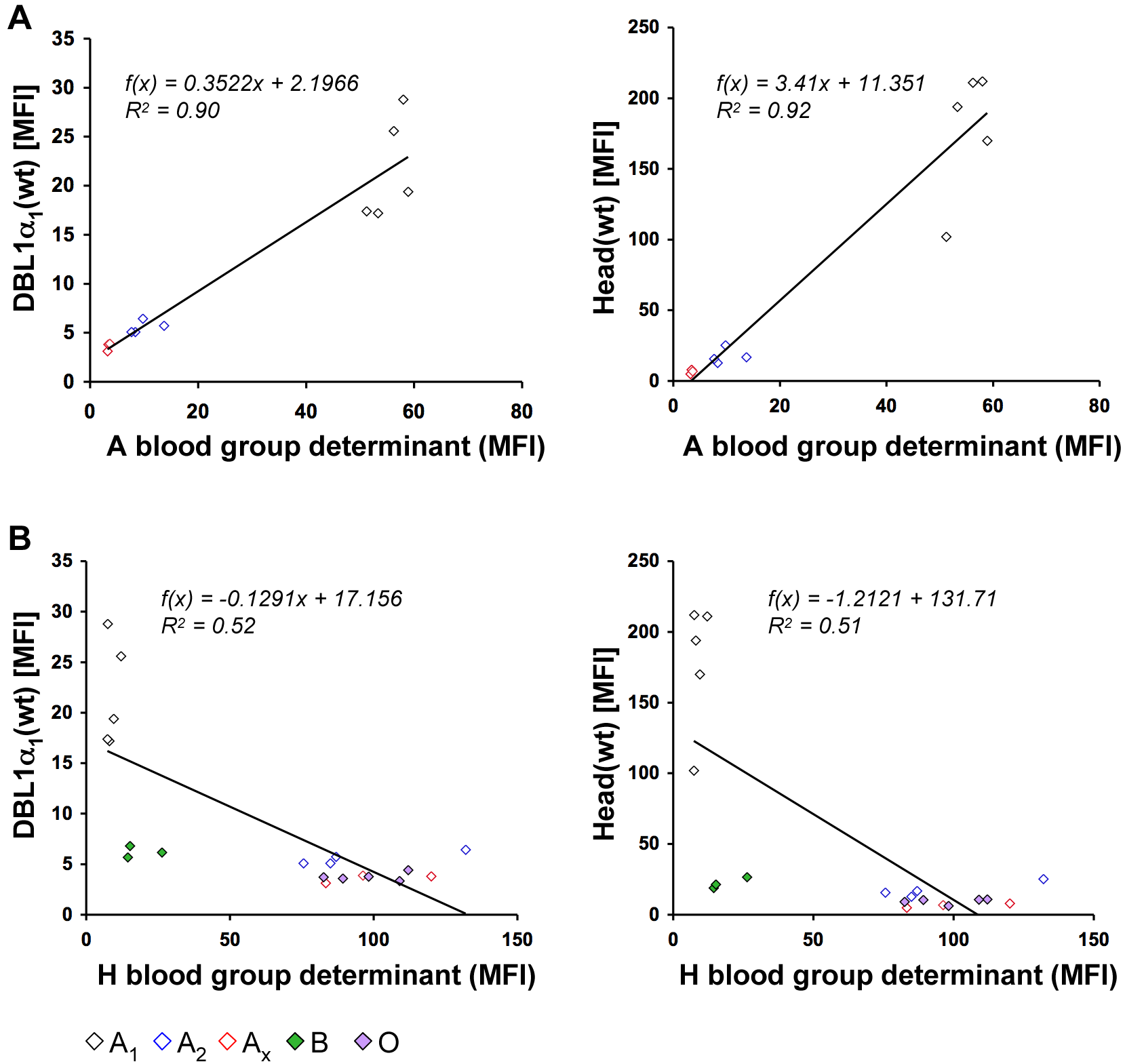

Supplement: Figure S6 — Relationship between RBC binding to DBL1α1(wt)and the Head(wt) and amount of A or H antigen displayed on the RBC. Graphs represented the linear regression plots of the association between the binding level [Mean Fluorescence Intensity (MFI) recorded by flow cytometry] of DBL1α1(wt) or Head(wt) and blood group A or H antigen expression levels. (A) Association between binding levels on A RBC subgroups (A1, A2 and Ax) and blood group A antigen expression. (B) Association between binding levels on A1, A2, Ax, B and O RBC and H antigen expression. For each graph, the regression equation and the coefficient of determination (R2) are shown. (TIF) [file ppat.1002781.s006.tif]

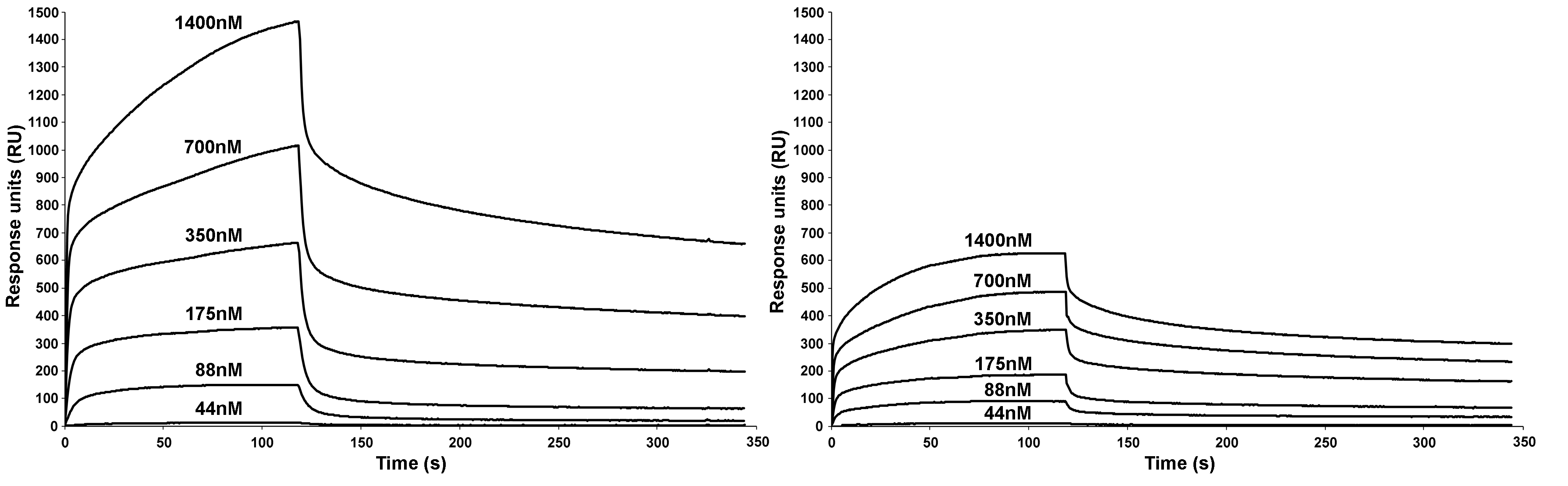

Supplement: Figure S7 — Binding of Head(wt) to BSA-conjugated A or B trisaccharide. Real-time association and dissociation profiles monitored by surface plasmon resonance, corresponding to the injection of different concentrations of the Head(wt)domain, over immobilized BSA-conjugated trisaccharide A (left) or trisaccharide B (right). (TIF) [file ppat.1002781.s007.tif]

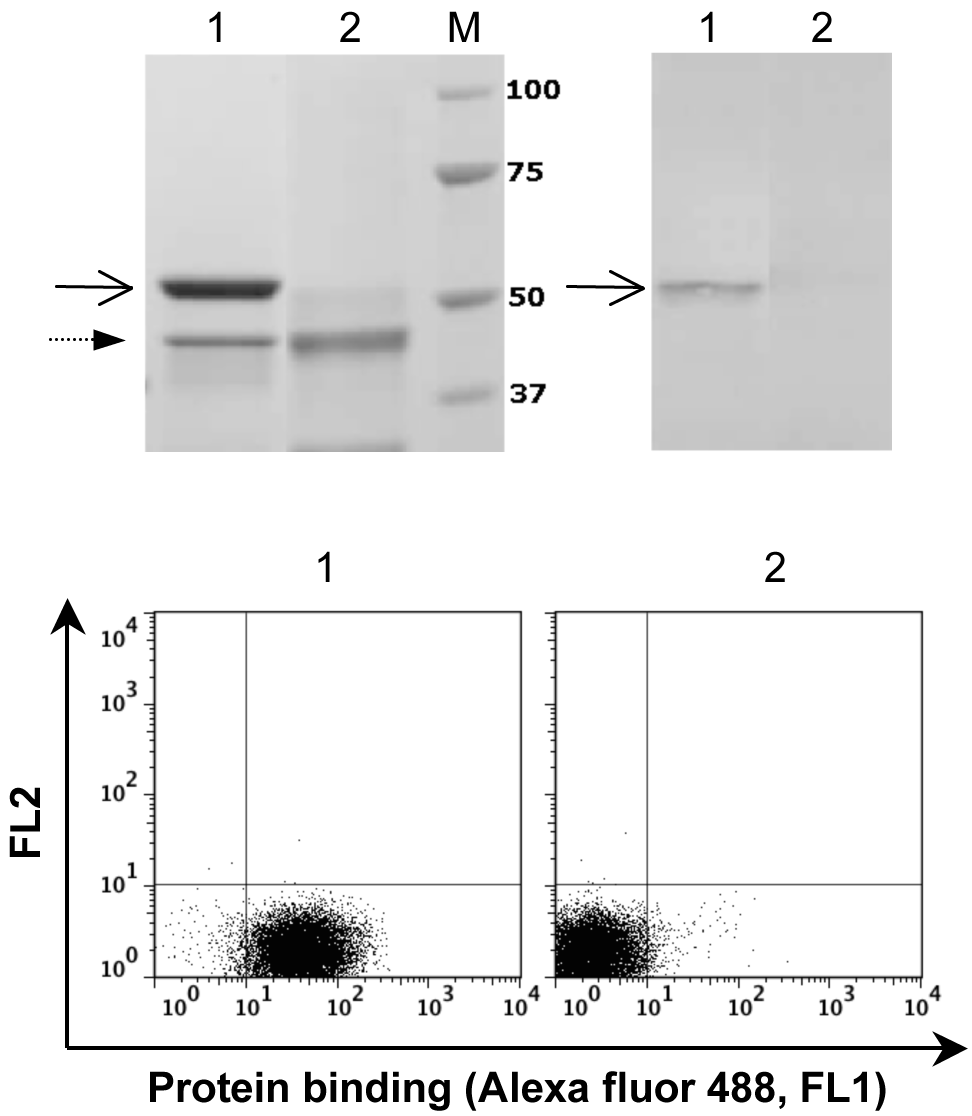

Supplement: Figure S8 — Recombinant DBL1α1 domains cleaved after R64 or R69 no longer bind RBC. The left panel shows a Coomassie Blue R-stained SDS-PAGE gel, with evidence of cleavage of DBL1α1(lane 1), which migrated as two bands. N-terminal sequencing showed that the lower band (dotted arrow) started at residue N70SDG (i.e. the protein was cleaved after R69). Lane 2 shows a factor Xa-cleaved Mut10, in which an internal factor Xa cleavage has been inserted at positions 66–69 (R64YGYVR69 mutated to R64YIEGR69), and the domain released from its MBP carrier by thrombin cleavage (the plasmid was engineered to create a thrombin cleavage site after the MBP coding sequence). The proteins were used in RBC binding assays and binding was visualised by immunoblot to identify the RBC-bound band(s) (right panel), DBL1α1 (lane 1), cleaved Mut10 (lane 2) or flow cytometry - DBL1α1(panel 1), cleaved Mut10 (panel 2). (TIF) [file ppat.1002781.s008.tif]

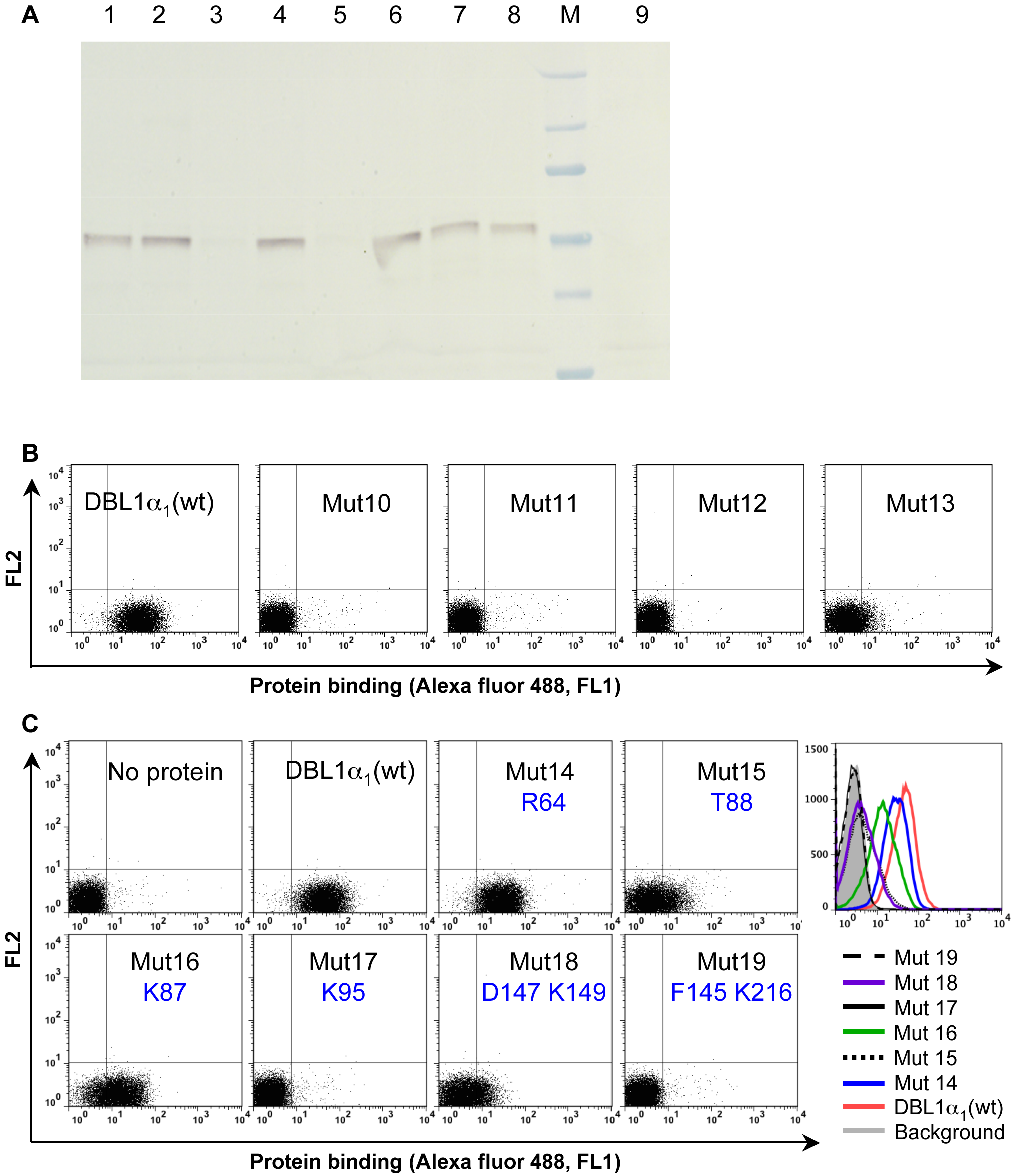

Supplement: Figure S9 — Binding characteristics of the mutantdomains. (A) Immunoblot of a typical RBC assay with Mut0 (lane 1), Mut1 (lane 2), Mut2 (lane 3), Mut3 (lane 4), Mut4 (lane 5), Mut5 (lane 6), Mut6 (lane 7), Mut7 (lane 8) and Mut10 (lane 9) probed with a polyclonal mouse anti-DBL1α1antiserum. M; molecular mass markers. (B) Dot plot representation of flow cytometry analysis of DBL1α1(wt) and Mut10-13 binding to A1 blood group RBCs. (C) Dot plot representation of flow cytometry analysis of Mut14-19 constructed on the DBL1α1(wt) background. Protein binding was probed with a polyclonal mouse anti-DBL1α1antiserum. The histogram on the right shows the distribution of fluorescence intensity for each mutant, colour-coded as indicated. Representative results of three independent assays. (TIF) [file ppat.1002781.s009.tif]

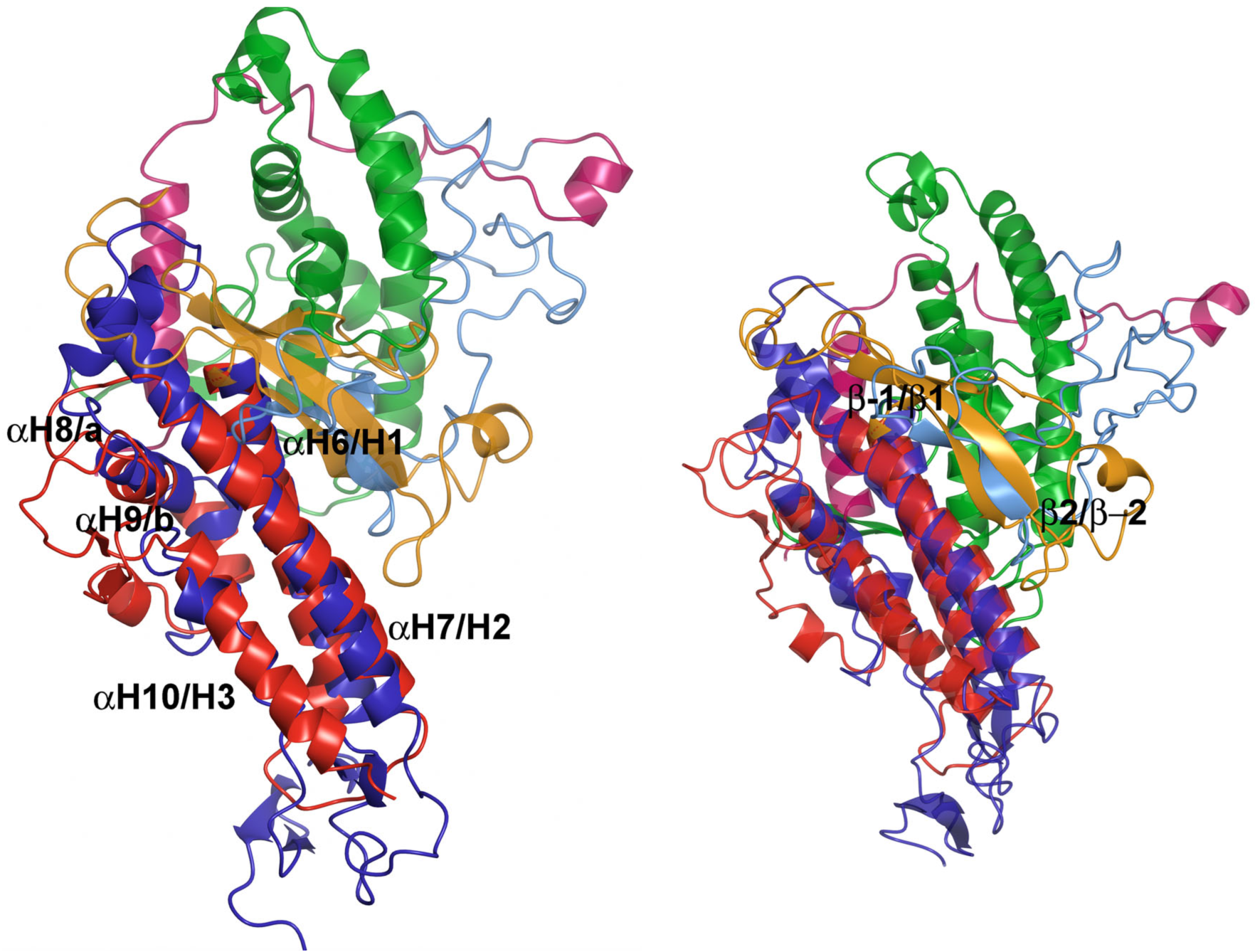

Supplement: Figure S10 — Superposition of CIDR1γ upon DBL1α1. Superposition of the domains showing correspondence of helices (left) and β strands (right). Equivalent secondary structure elements are labelled (DBL1α1 followed by CIDR1γ). The domains are shown in ribbon representation with the subdomain colour code as in Figures 5 and 6. (TIF) [file ppat.1002781.s010.tif]
