# Supplementary material for: Structural Basis for the ABO Blood-Group Dependence of Plasmodium falciparum Rosetting
Source: PLoS Pathog. 2012 Jul 12;8(7):e1002781. doi: 10.1371/journal.ppat.1002781 (PMC3395597; doi:10.1371/journal.ppat.1002781)
Supplement: Table S1 — Analysis of antibodies induced by the recombinant domains in outbred OF1 mice. (DOC) [file ppat.1002781.s011.doc]

**Table S1**

**Analysis of antibodies induced by the recombinant domains in outbred OF1 mice**

| Proteins | Endpoint titers (ELISA) ^#^ | Western blot (VarO-iRBCs)^§^ | | Surface reactivity (Flow cytometry) ** | Rosette dissociation ** |
| --- | --- | --- | --- | --- | --- |
|  |  | Non-reduced | Reduced |  |  |
| DBL1α_1_/DBL1α _1_(wt) | 1 / 5 x10^6^ | + | + | + | + |
| Head /Head(wt) | 1 / 5 x10^6^ | + | + | + | + |
| CIDR1γ* | 1 / 5 x10^5^ | + | + | + | - |
| DBL2β* | 1 / 10^6^ | + | - | + | - |
| DBL3γ* | 1 / 5 x10^5^ | + | + | - | - |
| DBL4ε* | 1 / 5 x10^5^ | + | + | + | - |
| DBL5ε | 1 / 2 x10^6^ | + | +/- | - | - |

* Domains with predicted N-glycosylation sites mutated.

^#^ Antibody titers (end-point titer) were determined by ELISA on the cognate antigen.

^§^ Immunoblot reactivity on Palo Alto 89F5 VarO parasite extracts was assessed with a 1/100 serum dilution.

** Reactivity with the Palo Alto 89F5 VarO-iRBC surface and rosette dissociation capacity were assessed using a 1/20 serum dilution.
