# Supplementary material for: Structural Basis for the ABO Blood-Group Dependence of Plasmodium falciparum Rosetting
Source: PLoS Pathog. 2012 Jul 12;8(7):e1002781. doi: 10.1371/journal.ppat.1002781 (PMC3395597; doi:10.1371/journal.ppat.1002781)
Supplement: Table S2 — CIDR1γ domain disulfide bonds. (DOC) [file ppat.1002781.s012.doc]

**Table S2**

**CIDR1γ domain disulfide bonds**

| **Canonical Cys number** | **Position in sequence** |
| --- | --- |
| Cys(1) - Cys(8) | C534 - C614 |
| Cys(2) - Cys(3) | C546 - C555 |
| Cys(4) - Cys(6) | C597 - C608 |
| Cys(7) - Cys(9) | C612 - C710 |
| Cys(8a) - Cys(8b) | C678 - C684 |
