# Supplementary material for: Structural Basis for the ABO Blood-Group Dependence of Plasmodium falciparum Rosetting
Source: PLoS Pathog. 2012 Jul 12;8(7):e1002781. doi: 10.1371/journal.ppat.1002781 (PMC3395597; doi:10.1371/journal.ppat.1002781)
Supplement: Table S3 — Sedimentation coefficient (S20,w) and frictional ratio (f/f0) of the recombinant domains. (DOC) [file ppat.1002781.s013.doc]

**Table S3**

**Sedimentation coefficient (S_20,w_) and frictional ratio (f/f_0_) of the recombinant domains**

| ***Protein*** |  | ***S*** | ***f/f0*** |  | ***S Hydropro**** | ***S US-SOMO**** |
| --- | --- | --- | --- | --- | --- | --- |
| **DBL1α_1_** |  | 3.8 ± 0.1 | 1.40 ± 0.03 |  | 3.9 | 3.9 |
| **CIDRγ** |  | 2.4 ± 0.1 | 1.35 ± 0.1 |  | 2.4 | 2.4 |
| **DBL1α_1_-CIDR1γ** |  | 5.2 ± 0.1 | 1.31 ± 0.08 |  | 5.2 | 5.3 |

* Sedimentation coefficients calculated from crystal coordinates.
